# Immunomodulatory Effects of (*R*)-Sulforaphane on LPS-Activated Murine Immune Cells: Molecular Signaling Pathways and Epigenetic Changes in Histone Markers

**DOI:** 10.3390/ph15080966

**Published:** 2022-08-04

**Authors:** Manuel Alcarranza, Isabel Villegas, Rocío Muñoz-García, Rocío Recio, Inmaculada Fernández, Catalina Alarcón-de-la-Lastra

**Affiliations:** 1 Department of Pharmacology, Faculty of Pharmacy, Universidad de Sevilla, 41012 Sevilla, Spain; 2 Departamento de Química Orgánica y Farmacéutica, Facultad de Farmacia, Universidad de Sevilla, 41012 Sevilla, Spain

**Keywords:** antioxidant, epigenetic, histone, inflammation, macrophages, (*R*)-sulforaphane, spleen cells

## Abstract

The aim of this study was to explore the immunomodulatory effects of the natural enantiomer (*R*)-Sulforaphane (SFN) and the possible signaling pathways involved in an ex vivo model of LPS-stimulated murine peritoneal macrophages. Furthermore, we studied the epigenetic changes induced by (*R*)-SFN as well as the post-translational modifications of histone H3 (H3K9me3 and H3K18ac) in relation to the production of cytokines in murine splenocytes after LPS stimulation. (*R*)-SFN was able to modulate the inflammatory response and oxidative stress induced by LPS stimulation in murine peritoneal macrophages through the inhibition of reactive oxygen species (ROS), nitric oxide (NO) and cytokine (IL-1β, IL-6, IL-17, IL-18 and TNF-α) production by down-regulating the expression of pro-inflammatory enzymes (iNOS, COX-2 and mPGES-1). We also found that activation of the Nrf-2/HO-1 axis and inhibition of the JAK2/STAT-3, MAPK, canonical and non-canonical inflammasome signaling pathways could have been responsible for the immunomodulatory effects of (*R*)-SFN. Furthermore, (*R*)-SFN modulated epigenetic modifications through histone methylation (H3K9me3) and deacetylation (H3K18ac) in LPS-activated spleen cells. Collectively, our results suggest that (*R*)-SFN could be a promising epinutraceutical compound for the management of immunoinflammatory diseases.

## 1. Introduction

Specific functional food supplements, such as sulforaphane (SFN)—an isothiocyanate compound—(1-isothiocyanate-(4*R*)-(methylsulfinyl)butane), are currently attracting a great amount of interest due to their high biological activity. Several vegetables of the cruciferous family, such as broccoli and cauliflower, among others, present SFN in the form of glucosinolate (glucoraphanin), which is hydrolyzed in part by the action of the enzyme myrosinase during cooking or chewing and by the gut microbiota after ingestion [1].

Available data indicate that the biological activities of natural isothiocyanates are defined by their structural characteristics, as it can be observed that small changes can induce important modifications in their effects. Another feature to take into account is the oxidation state of the sulfur atom, as this can modify the potency of the compound; for example, the sulfoxide derivative (sulforaphane) is more active than the thioether I and the sulfone II derivative (Figure S1) [2,3,4].

Even though two enantiomers are possible, the natural SFN exists as a single enantiomer with an *R_S_* absolute configuration (Figure S1) [5].

SFN is considered a natural antioxidant and free radical scavenger and is also a potent activator of the nuclear factor erythroid 2 (Nrf-2) signaling pathway [6]. In addition, SFN has shown interesting bioactivities as an antidiabetic, antihyperlipidaemic, antimicrobial, anticancer, neuro- and cardioprotective agent [7,8,9,10,11].

Some of the most important components of the immune system are the macrophages, which play a key role in the inflammatory process. In activated macrophages, the pathogen-associated molecular pattern, bacterial lipopolysaccharide (LPS)—an endotoxin re-leased from the external membrane of Gram-negative bacteria—is recognized by Toll-like receptor 4 (TLR4), committing macrophages to an inflammatory state and triggering the release of pro-inflammatory mediators, such as Th1 and Th17 cytokines and chemokines. These immune cells also change their mitochondrial metabolism by generating reactive oxygen species (ROS) and biosynthetic precursors through ATP synthesis via OXPHOS [12].

In addition, overexpression of pro-inflammatory enzymes, in particular inducible nitric oxide synthase (iNOS), cyclooxygenase (COX)-2 and microsomal prostaglandin E synthase (mPGES)-1, responsible for the synthesis of nitric oxide (NO) and prostaglandin (PG)E2, respectively, has also been documented in activated macrophages. Multiple signaling cascades are involved in the gene expression mechanism of these pro-inflammatory mediators, culminating in the activation of transcription factors, such as mitogen-activated protein kinases (MAPKs), nuclear transcription factor kappa B (NF-κB), Janus kinase/signal transducer and activator of transcription (JAK/STAT) or inflammasome activation [13,14,15].

Furthermore, the expression of antioxidant enzymes such as heme oxygenase-1 (HO-1) is regulated by Nrf-2, a redox-sensitive transcription factor. This signaling pathway is inhibited in activated macrophages due to stimulation by ROS, MAPKs and the inflammasome [16]. Therefore, LPS-activated murine macrophages represent an excellent model for evaluating new immunomodulators and anti-inflammatory drugs.

So far, few studies have been conducted on the importance of sulfur chirality in the biological activity of SFN [17]. In this regard, the initial data showed that sulfur chirality had no significant biological activity. However, recent research has shown that the natural (*R*)-SFN is a more potent enzymatic inducer of the lung and liver carcinogenic detoxification system in rats than the non-natural (*S*)-isomer [18]. A subsequent investigation of the regulation of two enzyme systems of carcinogen metabolism, epoxide hydrolase and glucuronosyl transferase, showed that (*R*)-SFN increased microsomal epoxide hydrolase levels more than the (*S*)-isomer. On the other hand, (*R*)-isomer increased glucuronosyl transferase activity, with the opposite effect observed with (*S*)-SFN [19]. Despite these data, naturally occurring SFN exists as a unique enantiomer, although most studies focusing on its biological activities, in particular its anti-inflammatory and antioxidant activities, have been conducted using its racemic (*rac*) form. In fact, scientific evidence indicates that *rac*-SFN has shown these effects in several in vitro [20,21,22,23] and in vivo models [24,25,26,27].

In the present work, in order to obtain enantiomerically pure (*R*)-SFN, we applied the “DAG methodology” [28,29]. This method has been developed in our group and it seems to be the methodology of choice for various reasons, such as being able to obtain a diasteromerically pure sulfinate ester with a non-hindered alkyl chain at the sulfinyl sulfur, and by a simple change of the base used to catalyse the reaction, it can make both epimers at sulfur accessible in an enantiodivergent manner through a dynamic kinetic transformation of the starting sulfinyl chloride (Figure S2) [30,31].

**Figure 1:**
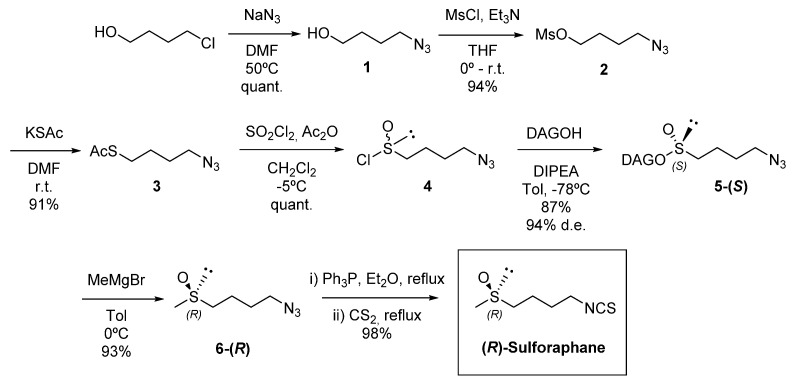
Enantioselective synthesis of (*R*)-sulforaphane by the DAG methodology.

In fact, the DAG methodology has allowed us to synthesize enantiopure (*R*)-SFN in just seven reaction steps, with excellent yields (68% overall yield), from inexpensive starting materials (Figure 1) [32,33,34,35,36].

Nutritional compounds are capable of modulating pathological processes, such as inflammation, by regulating gene expression without changing the genetic code, through key epigenetic mechanisms, e.g., histone acetylation/deacetylation, small non-coding RNA action and DNA methylation, as reported with recent data. Their ability to change epigenetic patterns depends directly on the interaction with enzymes that add or suppress epigenetic markers, or indirectly on the normalization of proteins encoded by genes involved in the epigenetic system [37].

Recent studies suggest that epigenetic changes, in particular histone modifications, play an important role in immune system tolerance, preventing damage by excessive inflammatory response. Indeed, Lauterbach et al. have shown that macrophages adopt their metabolism upon LPS activation by promoting histone acetylation [12].

It has been documented that *rac*-SFN positively influences the acetylation/deacetylation balance of histones in chromatin. The epigenetic control of histone deacetylase (HDAC) activity has clearly explained the molecular mode of action of this isothiocyanate. In fact, *rac*-SFN has been identified as an inhibitor of HDAC not only in cancer cells, but also in dendritic cells, cortical neurons and neural crest cells [38]. Moreover, this isothiocyanate has also been considered as a potential regulator of DNA methylation in the development of cancer and hypertension [39,40]. In addition, *rac*-SFN was also reported to modulate miRNA expression in colorectal cells [41]. However, the role of the natural enantiomer (*R*)-SFN as an epigenetic modulator in the inflammatory response generated by LPS is unknown.

Although the bioactivity of the *rac*-SFN compound has been previously reported in a wide variety of experimental models, as discussed above, the pharmacological effects of the natural isothiocyanate have not yet been investigated. In this context, the main objective of the present study was to evaluate the anti-inflammatory and antioxidant activity of (*R*)-SFN in an ex vivo model of inflammation using LPS-stimulated murine peritoneal macrophages in order to initially validate its future use as a nutraceutical compound. In particular, we aimed to characterize the mechanism of action and signaling pathways possibly involved in the potentially beneficial effects of (*R*)-SFN. Finally, to explore the (*R*)-SFN-induced epigenetic changes involved, we studied histone H3 post-translational modifications (H3K9me3, H3K27me3 and H3K18ac) and related cytokine production in spleen cells after LPS stimulation.

## 2. Results

### 2.1. Effect of (R)-SFN on the Viability of Murine Peritoneal Macrophages

**Figure 2:**
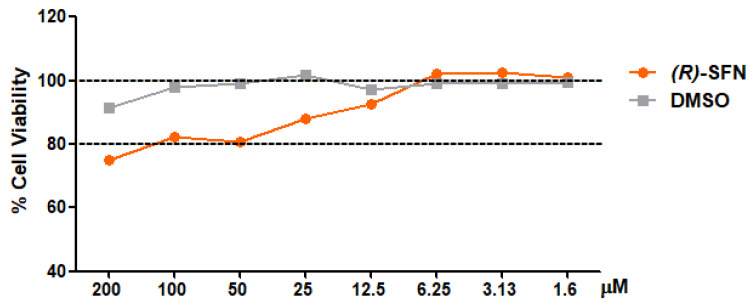
Effect of (*R*)-SFN on cell viability. Cells were treated with (*R*)-SFN (200–1.6 µM) for 18 h. Cell survival was represented as the percentage of viability with respect to 100% of untreated control cells.

The cytotoxic effects of (*R*)-SFN on peritoneal macrophages was determined using the SRB (sulforhodamine B) assay. Cells were treated with various concentrations of (*R*)-SFN (200–1.6 μM) for 18 hours (h). (*R*)-SFN did not compromise cell viability (≥80%) at doses of 100–1.6 μM. The vehicle (dimethylsulfoxide, DMSO) also had no impact on cell viability (Figure 2).

### 2.2. (R)-SFN Down-Regulated iNOS, Nitrite Production and Intracellular ROS in LPS-Stimulated Murine Peritoneal Macrophages

**Figure 3:**
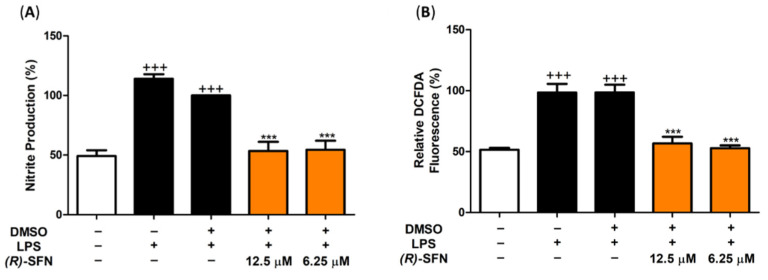

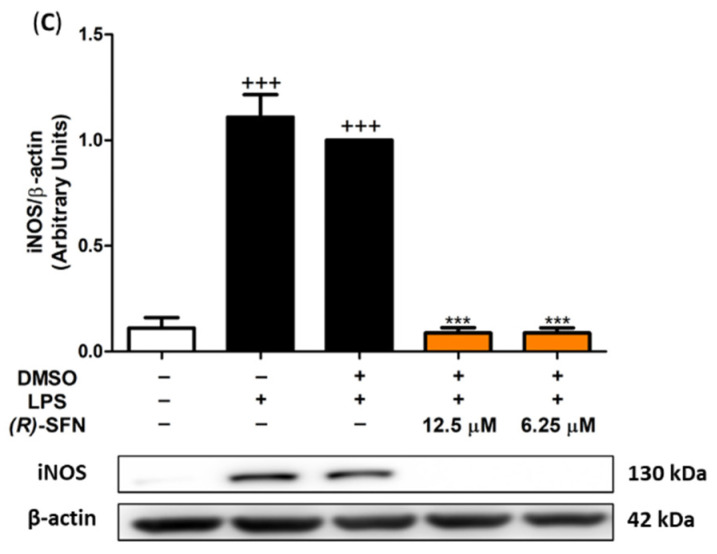
Effects of (*R*)-SFN on NO (**A**) and ROS (**B**) production and iNOS protein expression (**C**). Cells were treated with (*R*)-SFN (12.5 and 6.25 µM) for 30 min (min) and then exposed to 5 µg/mL of LPS for 18 h. The β-actin housekeeping gene was used as a control to normalize the densitometry performed. Data are expressed as means ± standard errors (SEMs) (*n* = 6). (+++) *p* < 0.001 vs. control cells (unstimulated); (***) *p* < 0.001 vs. LPS-DMSO-treated cells.

To gain insight into the effects of (*R*)-SFN on the LPS-induced oxidative response, intracellular NO and ROS levels, as well as iNOS expression, were determined using the Griess assay, a 2′,7′–dichlorofluorescin diacetate (DCFDA) kit and Western blotting, respectively. Figure 3 shows a significant increase in the DCFDA fluorescence signal in LPS-DMSO-induced cells as a positive control (+++ *p* < 0.001 vs. unstimulated control cells). However, pretreatment with (*R*)-SFN was able to significantly attenuate ROS production, demonstrating potent antioxidant activity (*** *p* < 0.001 vs. LPS-DMSO cells) (Figure 3). When murine peritoneal macrophages are exposed to LPS, NO is synthesized and released into the extracellular milieu via iNOS protein expression. Due to the low half-life of NO, nitrites were measured as an indicator of NO synthesis levels. LPS activation resulted in a statistically significant increase in NO production levels compared to the unstimulated control group (+++ *p* < 0.001 vs. control cells (unstimulated)). As shown in Figure 3, pretreatment with 12.5 and 6.25 µM of (*R*)-SFN significantly reduced NO generation, which was confirmed by a reduction in iNOS expression (*** *p* < 0.001 vs. LPS-DMSO-treated cells).

### 2.3. Effects of (R)-SFN on TNF-α, IL-1β, IL-6 and IL-17 Production

**Figure 4:**
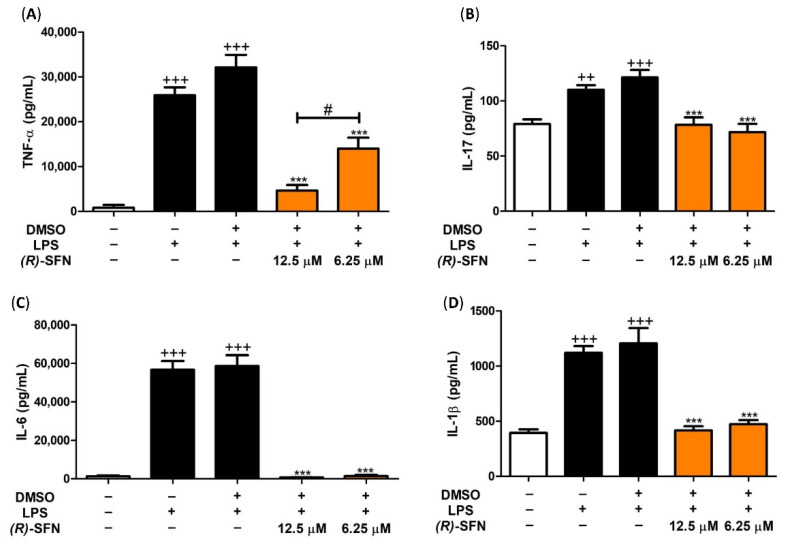
Treatment with (*R*)-SFN decreased the production of TNF-α (**A**), IL-17 (**B**), IL-6 (**C**) and IL-1β (**D**) levels. Murine peritoneal macrophages were treated with (*R*)-SFN (12.5 or 6.25 µM) 30 min before stimulation with LPS (5 µg/mL) for 18 h, then cytokine secretion was analyzed by enzyme-linked immunosorbent assay (ELISA) in cell supernatants. Data are expressed as means ± SEMs (*n* = 8). (++) *p* < 0.01; (+++) *p* < 0.001 vs. control cells (unstimulated); (***) *p* < 0.001 vs. LPS-DMSO-treated cells; (#) *p* < 0.05 vs. cells treated with 6.25 µM (*R*)-SFN.

To evaluate the effects of (*R*)-SFN on the production of pro-inflammatory cytokines, we measured the levels of TNF-α, IL-1β, IL-6 and IL-17 in peritoneal macrophage culture supernatants. Figure 4 shows a significant increase in TNF-α, IL-1β, IL-6 and IL-17 production after stimulation with LPS for 18 h compared to unstimulated control cells (+++ *p* < 0.001 vs. unstimulated cells). In contrast, after pretreatment with 12.5 and 6.25 µM of (*R*)-SFN, the levels of TNF-α, IL-1β, IL-6 and IL-17 were significantly reduced compared to the LPS-DMSO group (*** *p* < 0.001 vs. LPS-DMSO-stimulated cells).

### 2.4. (R)-SFN Decreased LPS-Induced COX-2 and mPGES-1 Overexpression

**Figure 5:**
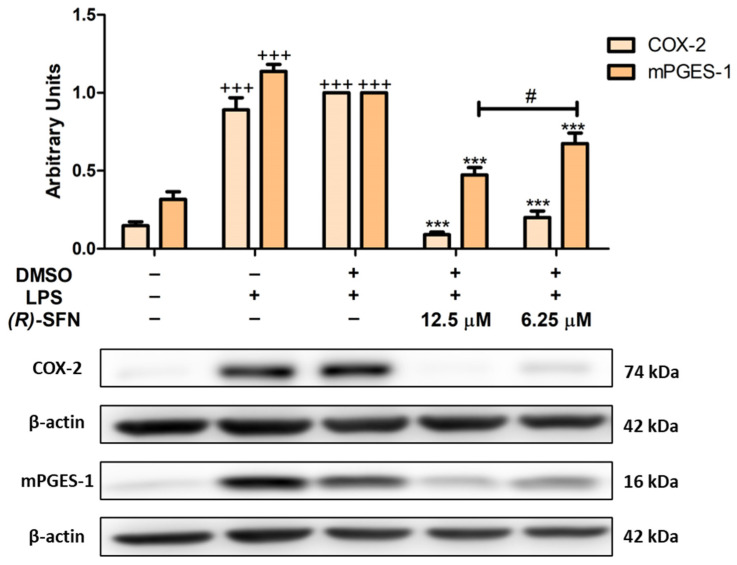
(*R*)-SFN decreased COX-2 and mPGES-1 expression in murine peritoneal macrophages. Cells were treated with (*R*)-SFN (12.5 and 6.25 µM) for 30 min and then exposed to 5 µg/mL of LPS for 18 h. Histograms show the densitometric analysis, normalized to the β-actin housekeeping gene of COX-2 or mPGES-1 proteins. Data are represented as means ± SEMs (*n* = 6). (+++) *p* < 0.001 vs. un-stimulated control cells; (***) *p* < 0.001 vs. LPS-DMSO-treated cells; (#) *p* < 0.05 vs. cells treated with 6.25 µM (*R*)-SFN.

To evaluate the anti-inflammatory effects of (*R*)-SFN, we analyzed the inflammation-related biomarkers COX-2 and mPGES-1. After LPS stimulation, COX-2 and mPGES-1 protein expression was significantly increased in LPS-DMSO control cells compared to unstimulated cells (+++ *p* < 0.001 vs. unstimulated control cells). However, (*R*)-SFN pretreatment (6.25 and 12.5 µM) down-regulated COX-2 and mPGES-1 protein expression (*** *p* < 0.001 vs. LPS-DMSO-treated cells). Interestingly, 12.5 µM of (*R*)-SFN reduced mPGES-1 expression significantly more effectively than 6.25 µM (# *p* < 0.05 vs. cells treated with 6.25 µM (*R*)-SFN) (Figure 5).

### 2.5. (R)-SFN Up-Regulated Nrf-2/HO-1 Axis Protein Expression in LPS-Stimulated Murine Peritoneal Macrophages

**Figure 6:**
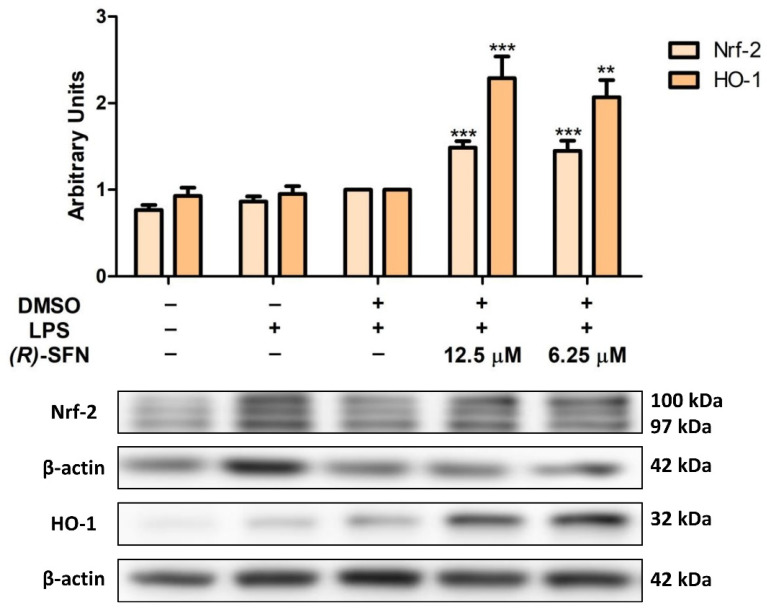
(*R*)-SFN increased the expression of Nrf-2 and HO-1 in murine peritoneal macrophages activated by LPS. Murine immune cells were treated with (*R*)-SFN for 30 min, followed by stimulation with LPS for 18 h. Histograms show the densitometric analysis of Nrf-2 and HO-1 proteins normalized to the β-actin housekeeping gene. Data are expressed as means ± SEMs (*n* = 6). (**) *p* < 0.01; (***) *p* < 0.001 vs. LPS-DMSO-treated cells.

Western blotting was used to determine whether (*R*)-SFN could regulate the antioxidant Nrf-2/HO-1 signaling pathway. Treatment with (*R*)-SFN produced a significant up-regulation in the expression of both proteins in comparison to the control LPS-DMSO cells (** *p* < 0.01; *** *p* < 0.001 vs. LPS-DMSO-treated cells) (Figure 6). These results, together with the decrease in ROS production, could demonstrate the significant antioxidant activity of (*R*)-SFN in this preclinical model.

### 2.6. Effects of (R)-SFN on MAPKs Activation in LPS-Activated Peritoneal Macrophages

**Figure 7:**
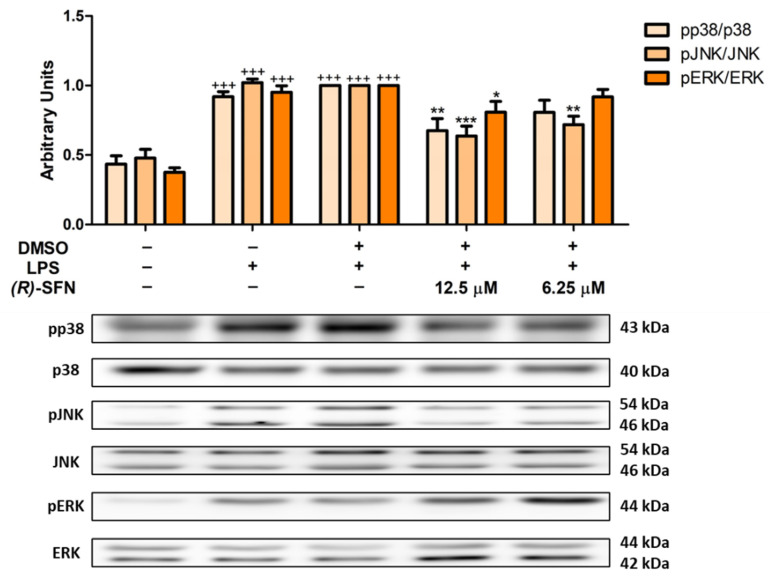
(*R*)-SFN decreased the phosphorylation of p38, JNK and ERK in LPS-activated peritoneal macrophages. Cells were pretreated with (*R*)-SFN (12.5 and 6.25 µM) for 30 min followed by LPS stimulation for 18 h. As controls, cells were also treated with DMSO (solvent control) and LPS or untreated in the absence of LPS. Histograms show the densitometric analysis of pp38, pJNK and pERK proteins normalized to the p38, JNK and ERK housekeeping gene, respectively. Data are expressed as means ± SEMs (*n* = 6). (+++) *p* < 0.001 vs. unstimulated control cells; (*) *p* < 0.05; (**) *p* < 0.01; (***) *p* < 0.001 vs. LPS-DMSO-treated cells.

We also evaluated the role of (*R*)-SFN on the activation of MAPKs by Western blotting. As shown in Figure 7, both concentrations (12.5 and 6.25 µM) were able to significantly reduce JNK phosphorylation (** *p* < 0.01; *** *p* < 0.001 vs. LPS-DMSO-treated cells), while only the higher concentration of (*R*)-SFN (12.5 µM) significantly attenuated the phosphorylation of ERK and p38 MAPKs (* *p* < 0.05; ** *p* < 0.01 vs. LPS-DMSO-treated cells).

### 2.7. Effects of (R)-SFN on the JAK2/STAT-3 Signaling Pathway in LPS-Activated Peritoneal Macrophages

The JAK/STAT signaling pathway plays an important role in the inflammatory response. Under conditions of cellular stress, phosphorylation of JAK2 is induced, which in turn allows phosphorylation of STAT, which is released from the receptor-binding complex and translocated to the cell nucleus to regulate the transcription of pro-inflammatory genes, including inducible enzymes or cytokines [42].

**Figure 8:**
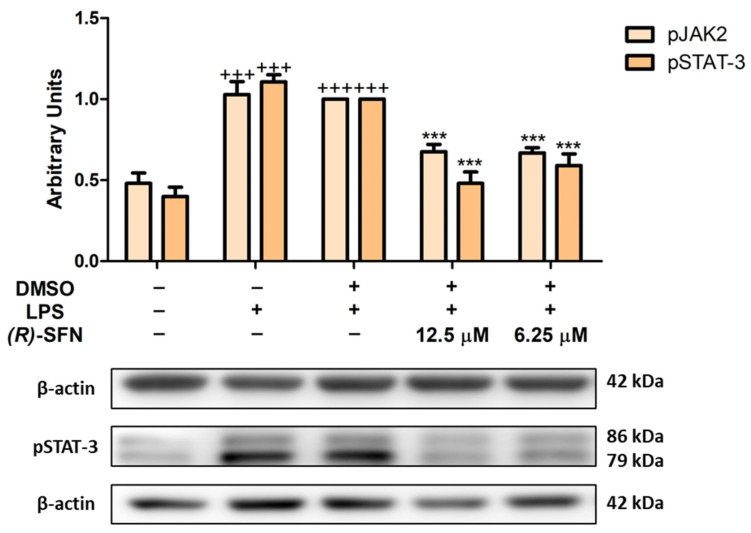
(*R*)-SFN reduced the phosphorylation of the JAK2/STAT-3 pathway in LPS-activated peritoneal macrophages. Murine peritoneal macrophages were treated with ®-SFN (12.5 and 6.25 µM) for 30 min, followed by LPS stimulation for 18 h. Histograms show the densitometric analysis of pJAK2 and pSTAT-3 proteins normalized to the β-actin housekeeping gene. Data are expressed as means ± SEMs (*n* = 6). (+++) *p* < 0.001 vs. unstimulated control cells; (***) *p* < 0.001 vs. LPS-DMSO-treated cells.

As can be seen in Figure 8, LPS treatment induced phosphorylation of JAK2 and STAT-3 in murine peritoneal macrophages (+++ *p* < 0.001 vs. unstimulated control cells); however, (*R*)-SFN treatments were able to significantly reduce their phosphorylation (*** *p* < 0.001 vs. LPS-DMSO-treated cells).

### 2.8. (R)-SFN Inhibited the Canonical and Non-Canonical Inflammasome in LPS-Activated Murine Peritoneal Macrophages

The domain-like receptor protein 3 (NLRP3) inflammasome is a multiprotein com-plex composed of a NLRP3 sensor molecule, an adaptor caspase recruitment protein (ASC) and pro-caspase 1. In the presence of inflammatory stimuli, such as LPS in murine peritoneal macrophages, activation of this complex occurs, producing the maturation of pro-caspase 1 to caspase 1, leading to the generation of IL-1β and IL-18 due to the activation of pro-IL-1β and pro-IL-18 cytokine precursors. In addition, the non-canonical pathway is also activated by LPS, which triggers the activation of pro-caspase 11 to caspase 11 and, consequently, the generation of IL-1β and IL-18 maturation, as already mentioned [43]. Accordingly, the effects of (*R*)-SFN on canonical and non-canonical activation of the inflammasome were determined by Western blotting.

**Figure 9:**
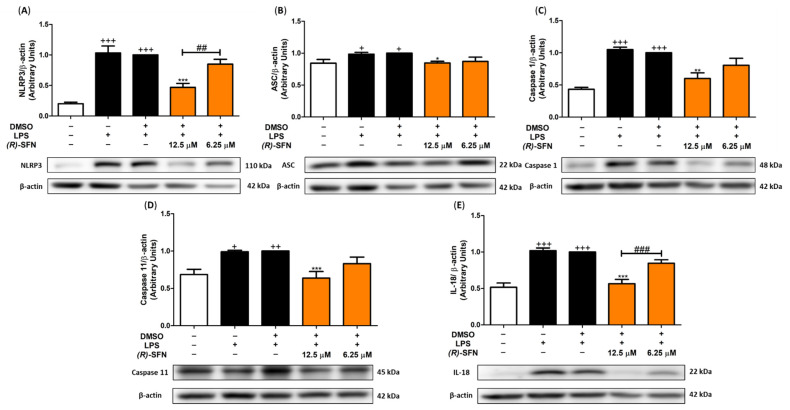
(*R*)-SFN down-regulated the inflammasome signaling pathway in murine peritoneal macrophages. Murine peritoneal macrophages were treated with (*R*)-SFN (12.5 and 6.25 µM) for 30 min, followed by LPS stimulation for 18 h. Histograms show the densitometric analysis of NLRP3 (**A**), ASC (**B**), caspase 1 (**C**), caspase 11 (**D**) and IL-18 (**E**) proteins normalized to the β-actin housekeeping gene. Data are expressed as means ± SEMs (*n* = 6). (+) *p* < 0.05; (++) *p* < 0.01; (+++) *p* < 0.001 vs. unstimulated control cells; (*) *p* < 0.05; (**) *p* < 0.01; (***) *p* < 0.001 vs. LPS-DMSO-treated cells; (##) *p* < 0.01; (###) *p* < 0.001 vs. cells treated with 6.25 µM (*R*)-SFN.

Figure 9 shows a significant increase in the protein expression of NLRP3, ASC, caspase 1 and caspase 11 in LPS-stimulated cells (+ *p* < 0.05; ++ *p* < 0.01; +++ *p* < 0.001 vs. unstimulated control cells). However, only the highest dose of (*R*)-SFN (12.5 µM) was able to significantly reduce the protein expression levels of NLRP3 (Figure 9A), ASC (Figure 9B), caspase 1 (Figure 9C) and caspase 11 (Figure 9D) (* *p* < 0.05; ** *p* < 0.01; *** *p* < 0.001 vs. LPS-DMSO-treated cells).

Similarly, the production of the cytokines IL-1β (Figure 4D) and IL-18 (Figure 9E) was also evaluated. As expected, LPS stimulation increased cytokine generation (+++ *p* < 0.001 vs. unstimulated control cells). However, only the highest dose of (*R*)-SFN (12.5 µM) significantly reduced the production of both cytokines (*** *p* < 0.001 vs. LPS-DMSO-treated cells) (Figure 4D and Figure 9E).

### 2.9. (R)-SFN Produced Epigenetic Histone Modifications in Spleen Cells

Finally, in order to explain the role of (*R*)-SFN in post-translational modification in H3K27me3, H3K18ac and H3k9me3 histones and its correlated cytokine production, we evaluated epigenetic modifications in spleen cells by Western blotting.

**Figure 10:**
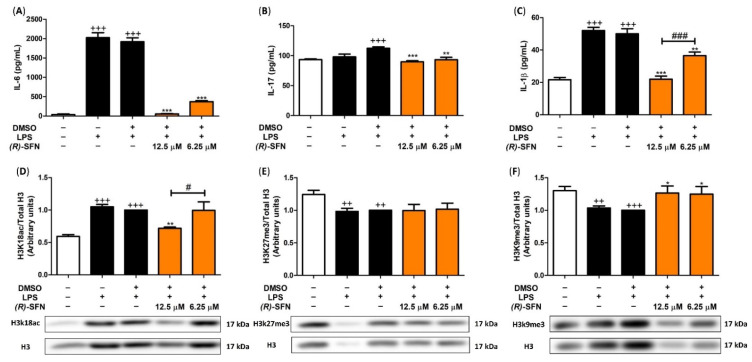
Effects of (*R*)-SFN on the production of IL-6 (**A**), IL-17 (**B**), IL-1β (**C**) and on the modification of the expression of H3 histones: H3K18ac (**D**), H3K27me3 (**E**) and H3K9me3 (**F**). Splenocytes were treated with (*R*)-SFN (12.5 and 6.25 μM) and stimulated with LPS for 24 h. Histones were isolated from spleen cells with acid extraction and were evaluated by Western blotting. The H3 housekeeping gene was used as a control to normalize the densitometry performed. Cytokine secretion was analyzed by ELISA in spleen cell supernatants. Results are presented as means ± SEMs (*n* = 6). (++) *p* < 0.01; (+++) *p* < 0.001 vs. unstimulated control cells; (*) *p* < 0.05; (**) *p* < 0.01; (***) *p* < 0.001 vs. LPS-DMSO-treated cells; (#) *p* < 0.05; (###) *p* < 0.001 vs. cells treated with (*R*)-SFN.

After LPS stimulation, H3K18ac levels were augmented (+++ *p* < 0.001 vs. unstimulated control cells) (Figure 10D), while the highest concentration of (*R*)-SFN (12.5 µM) significantly reduced H3 acetylation (** *p* < 0.01 vs. LPS-DMSO-treated cells).

In addition, the opposite behaviour was observed in histone methylation. In fact, the levels of H3K9me3 and H3k27me3 were significantly reduced when the spleen cells were stimulated with LPS (++ *p* < 0.01; +++ *p* < 0.001 vs. unstimulated control cells) (Figure 10E,F); however, the treatment with (*R*)-SFN only statistically raised the H3k9me3 expression (* *p* < 0.05 vs. LPS-DMSO-treated cells), while the H3k27me3 immunosignal remained unchanged.

As shown in Figure 10A–C, after LPS stimulation, IL-6, IL-1β and IL-17 cytokine levels were significantly increased (+++ *p* < 0.001 vs. unstimulated control cells). However, in splenocytes treated with (*R*)-SFN (12.5 and 6.25 µM), the production of these cytokines was statistically reduced (** *p* < 0.01; *** *p* < 0.001 vs. LPS-DMSO-treated cells). Notably, IL-1β levels were statistically more down-regulated with the highest concentration of (*R*)-SFN (12.5 µM) (### *p* < 0.001 vs. cells treated with (*R*)-SFN).

## 3. Discussion

The results of our study confirmed that the natural enantiomer (*R*)-SFN was able to induce immunomodulatory effects in LPS-activated murine peritoneal macrophages. These data are in agreement with the only study we have located in the literature that showed immunomodulatory activities of (*R*)-SFN in LPS-stimulated peripheral blood mononuclear cells (PBMCs) from healthy adults [44].

LPS-stimulated macrophages alter the intracellular redox balance, leading to the production of ROS. This plays a critical role in the pathogenesis of oxidative stress-related diseases. LPS induces ROS expression through several mechanisms, such as activation of NADPH oxidase and inhibition of antioxidant enzymes. Upon LPS stimulation, iNOS expression increases and high levels of NO are produced, which acts as an intracellular messenger modulating ROS production [45]. Interestingly, our results showed that (*R*)-SFN treatment was not only able to decrease these oxidation-related mediators, but that it also reduced intracellular ROS generation. Ranaweera et al. obtained similar results with *rac*-SFN by inhibiting LPS-induced ROS, NO and iNOS production in RAW 264.7 cells [23].

LPS-mediated activation is closely related to an imbalance of the cytokine network leading to the production of pro-inflammatory Th1 and Th17 cytokines, including IL-6, IL-1β, TNF-α and IL-17, among others [46,47]. Consistent with these observations, our data showed a marked increase in pro-inflammatory cytokine expression following LPS stimulation. However, treatment with (*R*)-SFN significantly decreased the secretion of the cytokines IL-1β, IL-6, TNF-α, IL-18 and IL-17. Similar data have been reported after treatment with the racemic compound in LPS-stimulated RAW 264.7 macrophages [21,23] and after treatment with (*R*)-SFN in LPS-activated PBMC populations in healthy adult volunteers [44].

Exposure of peritoneal macrophages to LPS induces overexpression of the inducible COX isoform (COX-2) and mPGES-1, which are involved in the overproduction of PGE2 responsible for increased chemotaxis, blood flow and subsequent tissue dysfunction during inflammation through activation of the EP4 receptor [48]. In agreement with our previous reports by Montoya et al., LPS stimulation significantly increased protein expression of COX-2 and mPGES-1 [49,50]. However, (*R*)-SFN decreased the immunosignal of both proteins. In this regard, other studies have shown that *rac*-SFN was able to inhibit COX-2 production in LPS-stimulated RAW 264.7 macrophages [23,51].

In addition, in order to further investigate the mechanism of action involved in (*R*)-SFN immunomodulatory effects, we studied several intracellular signaling pathways. Nrf-2 is an important modulator of cellular detoxification responses and redox status and is able to respond by translocating to the nucleus and interacting with the specific antioxidant responsive element (ARE) present in the promoter gene encoding detoxifying and antioxidant proteins, such as HO-1. Indeed, this enzyme plays a cytoprotective role against oxidative stress and inflammation in LPS-activated macrophages. In line with previous studies, we found that the Nrf-2/HO-1 signaling pathway was down-regulated after LPS treatment [49,50].

SFN is a known activator of Nrf-2 that directly induces Nrf-2 translocation and accumulation in the nucleus and may be phosphorylated by the activation of multiple kinases that stimulate the expression of detoxification enzymes, such as HO-1 [52]. Our results indicated that (*R*)-SFN treatment significantly increased the expression of both Nrf-2/HO-1 proteins. Although HO-1 also influences the anti-inflammatory action of SFN independently of the Nrf-2 pathway [53], our findings suggest that activation of the Nrf-2/HO-1 signaling pathway could be partly responsible for the anti-inflammatory activity of (*R*)-SFN by reducing the expression of pro-inflammatory enzymes, in particular COX-2 and iNOS, and other pro-inflammatory mediators, including TNF-α, IL-6, IL-1β, PGE2 and NO. Similar results were obtained with *rac*-SFN in a murine model of LPS-induced acute lung injury inflammation [24] and in LPS-activated microglia [54].

Furthermore, we proceeded to explore other effector signals, such as the JAK2/STAT-3 and MAPK signaling pathways.

JAK2 is a cytoplasmic tyrosine kinase responsible for the activation of STAT-3 prior to its phosphorylation and dimerization. Phosphorylated STAT-3 is then translocated to the nucleus to initiate the transcription of target genes. The JAK/STAT axis is known to play an important role in cytokine receptor signaling [16]. Therefore, we studied the possible involvement of the JAK2/STAT-3 signaling pathway in the immunomodulatory effects of (*R*)-SFN. Consistent with our previous studies, incubation with LPS promoted the phosphorylation of STAT-3 proteins in murine peritoneal macrophages [49,55]. In contrast, (*R*)-SFN was able to reduce this phosphorylation. Thus, our results consistently demonstrated that the natural isothiocyanate could down-regulate inflammatory cytokine expression by inhibiting the JAK2/STAT-3 signaling pathway in LPS-activated cells.

As mentioned above, MAPKs play a key role in macrophage activation by LPS. They are a family of serin–threonine kinases, including c-Jun N-terminal kinases (JNKs), p38 and extracellular signal-regulated kinases (ERKs-1 and -2) [56]. In response to inflammatory and oxidative stress stimuli, MAPKs can modulate the JAK/STAT pathway by inducing the expression of multiple genes that together regulate the expression of inflammatory chemokines and cytokines [57]. Our results in agreement with those of our recent reports indicated that after the incubation of macrophages with LPS, phosphorylation of JNK, p38 and ERK was statistically increased [55]. However, (*R*)-SFN treatment counteracted the effects of LPS by significantly decreasing the activation of JNK, ERK and p38 MAPKs. These data suggest that inactivation of MAPKs by (*R*)-SFN may be partly responsible for the down-regulation of the Th1- and Th17-mediated inflammatory response. Similar results have been obtained with *rac*-SFN in other experimental models, such as primary mouse bone-marrow-derived macrophages (BMDMs), human THP-1-derived macrophages and primary human peripheral blood mononuclear cell-derived macrophages [57].

A key role in pathogen defense and cytosolic innate immune sensing is played by the inflammasomes—intracellular complexes that have been extensively studied. Regarding the NLR family, the pyrin domain-containing protein 3 (NLRP3) inflammasome comprises three elements: a sensor (NLRP3), an adaptor apoptosis-associated speck-like protein containing a CARD (ASC) and a zymogen (pro-caspase 1). “Canonical inflammasome” is a multimeric complex formed by the activation of NLRP3, allowing the recruitment of ASC, which couples with pro-caspase 1, leading to its activated form, caspase 1. This caspase is able to contribute to the maturation and secretion of IL-18 and IL-1β, inducing pyroptosis and inflammatory cell death [58]. The non-canonical pathway is another activation mechanism for NLRP3 complex formation. In murine macrophages, in the presence of LPS, caspase 11 matures, resulting in potassium efflux leading to NLRP3 activation and pyroptosis. Thus, mature caspase 11 cooperates with the canonical NLRP3 inflammasome [59].

Our previous reports have shown that LPS-induced inflammation leads to activation of the NLRP3 inflammasome in macrophages [49,55]. Accordingly, in our research, inflammation induced by LPS led to the activation of canonical and non-canonical inflammasomes, as characterized by an increased expression of NLRP3, ASC, caspase 1 and caspase 11 protein expression, which was accompanied by an enhancement of IL-18 and IL-1β production.

*Rac*-SFN has been shown to block multiple inflammasomes, such as the NLRP3 inflammasome [60], in in vivo animal models, including stroke [61], retinal vascular disorder [62], acute gout [63], peritonitis [64], non-alcoholic fatty liver disease [65], acute pancreatitis [66] and pulmonary arterial hypertension [67] models, and also in vitro studies using BMDMs differentiated from RAW 264.7 cells [68], a THP-1 human monocytic cell line [69] and microglia [70]. In all of these studies, the racemate showed beneficial activities against NLRP3 canonical inflammasome activation, decreasing IL-1β and NLRP3 expression levels and caspase 1 activity.

Surprisingly, we show for the first time in this investigation that (*R*)-SFN treatment suppressed the activation of both canonical and non-canonical inflammasome signaling pathways after LPS activation in murine peritoneal macrophages. Thus, we provide evidence confirming that (*R*)-SFN modulates inflammatory responses by inhibiting the NLRP3 inflammasome via caspase 1 and caspase 11.

Epigenetic aberrations, including DNA methylation, histone modifications and miRNA deregulation, play a pivotal role in the development of inflammation. Recent studies have suggested that H3 post-translational modifications could influence pro-inflammatory cytokine production. In particular, histone acetylation is generally associated with increased gene expression due to decreased histone–DNA interaction; consequently, chromatin opens up and DNA accessibility increases, inducing gene transcription. In fact, Zhao et al. have suggested that NLRP3 inflammasome activation depends on the level of histone H3 acetylation [71]. On the other hand, methylation of H3K9me3 and H3K27me3 generally leads to suppressed gene expression by closing chromatin [72]. Furthermore, interesting studies have shown that histone methyltransferases contribute synergistically to inflammasome activation, collaborating in the inflammasome-induced inflammatory response [73].

In agreement with our results, increased H3K18ac and decreased H3K9me3 and H3K27me3 could be involved in LPS-induction of pro-inflammatory cytokine production in spleen cells. Nonetheless, the treatment with (*R*)-SFN regulated both H3K18ac and H3K9me3 expression and subsequently controlled IL-6, IL-1β and IL-17 production.

To summarize, (*R*)-SFN was able to modulate the inflammatory response and oxidative stress induced by LPS stimulation in murine peritoneal macrophages through the activation of the Nrf-2/HO-1 axis and inhibition of JAK2/STAT-3, ERK, JNK, p38 MAPKs and canonical and non-canonical inflammasome signaling pathways. Finally, the treatment with (*R*)-SFN down-regulated H3K18ac and up-regulated H3K9me3 expression and subsequently reduced IL-6, IL-1β and IL-17 production in spleen cells.

## 4. Materials and Methods

### 4.1. Chemicals

#### 4.1.1. Reagents and Instruments

Oven-dried glassware and dried solvents were used for the reactions, which were run under an atmosphere of dry argon. Chemicals were purchased from commercial sources and were used without further purification. Compounds were detected by thin-layer chromatography (TLC), which analyses were carried out on silica gel GF254 (Merck) and charred with phosphomolybdic acid/EtOH. Merck 230–400 mesh silica gel was used for flash chromatography. Positive pressure of air was used to elute chromatographic columns, and eluents are given as volume-to-volume ratios (*v*/*v*). Optical rotations were determined with a PerkinElmer 341 polarimeter. Nuclear magnetic resonance (NMR) spectra were recorded with Bruker Avance 500 MHz spectrometers. Chemical shifts are reported in ppm, and coupling constants are reported in Hz. High-resolution mass spectra (HRMS) were recorded at the Centro de Investigación, Tecnología e Innovación of the University of Seville with a Kratos MS-80RFA 241-MC apparatus.

#### 4.1.2. Synthesis of 4-Azidobutan-1-ol (**1**) 

To a solution of 4-chloro-1-butanol (85%) (29.4 g, 230.27 mmol) in dry DMF (190 mL) under argon atmosphere, sodium azide (44.9 g, 690.81 mmol) was added. The reaction mixture was heated to 50 °C overnight. After completion of the reaction, water (150 mL) was added and extracted with CH_2_Cl_2_ twice. The resulting organic layers were dried over anhydrous Na_2_SO_4_, and the solvent was evaporated to give 26.5 g (230.25 mmol, quantitative yield) of **1** as a colourless oil, which was used without further purification [33,35]. R_f_ = 0.35 (EtOAc/Hexane, 2:1); ^1^H NMR (500 MHz, CDCl_3_), δ 3.66 (t, 2H, J = 6.1 Hz), 3.31 (t, 2H, J = 6.6 Hz), 1.75 (bs, 1H), 1.72–1.60 (m, 4H), 1.54 (s, 1H) ppm (Figure S3); ^13^C NMR (125 MHz, CDCl_3_) δ 62.3, 51.4, 29.9, 25.5 ppm (Figure S4); HRMS (FAB) calcd. for C_4_H_9_N_3_O (M)^+^ *m*/*e* 115.0746, found *m*/*e* 115.0746.

#### 4.1.3. Synthesis of 4-Azidobutyl Methanesulfonate (**2**) 

Methanesulfonyl chloride (22.7 mL, 293.8 mmol) was added dropwise to a solution of 4-azidobutan-1-ol **1** (26.0 g, 226.01 mmol) and Et_3_N (40.9 mL, 293.81 mmol) in dry THF (175 mL), under argon atmosphere and at 0 °C. The reaction mixture was quenched with saturated NH_4_Cl aqueous solution and extracted with CH_2_Cl_2_ twice, after stirring at room temperature for 2 h. Then, the combined organic layers were washed with saturated NaCl aqueous solution and dried over anhydrous Na_2_SO_4_. The solvent was evaporated to give 40.8 g (211.38 mmol, 94% yield) of **2** as a colourless oil, which was used without further purification [33,35]. R_f_ = 0.54 (EtOAc/Hexane, 1:2); ^1^H NMR (500 MHz, CDCl_3_) *δ* 4.24 (t, 2H, *J* = 6.3 Hz), 3.34 (t, 2H, *J* = 6.7 Hz), 3.00 (s, 3H), 1.86–1.80 (m, 2H), 1.73–1.68 (m, 2H) ppm (Figure S5); ^13^C NMR (125 MHz, CDCl_3_) *δ* 69.2, 50.8, 37.5, 26.5, 25.1 ppm (Figure S6); HRMS (FAB) calcd. for C_5_H_11_O_3_N_3_NaS (M+Na)^+^ *m*/*e* 216.0412, found *m*/*e* 216.0413.

#### 4.1.4. Synthesis of 4-Azidobutyl-1-thioacetate (**3**) 

To a solution of 4-azidobutyl methanesulfonate **2** (30.26 g, 156.61 mmol) in dry DMF (500 mL) under argon atmosphere, potassium thioacetate (23.3 g, 203.59 mmol) was added at room temperature. The reaction was left stirring overnight. Then, water was used to wash the mixture and EtOAc was used to extract it three times. NaHCO_3_ aqueous solution and brine were used to wash the combined organic phases. Afterwards, the product was dried over anhydrous Na_2_SO_4_ and evaporated to obtain 24.7 g (142.51 mmol, 91% yield) of **3** as a brown oil, which was used without further purification [33,35]. *R*_f_ = 0.65 (EtOAc/Hexane, 1:9); ^1^H NMR (500 MHz, CDCl_3_) *δ* 3.30–3.33 (m, 2H), 2.90–2.87 (m, 2H), 2.32 (s, 3H), 1.67–1.64 (m, 4H) ppm (Figure S7); ^13^C NMR (125 MHz, CDCl_3_) *δ* 195.8, 51.0, 30.7, 28.6, 28.0, 26.9 ppm (Figure S8); HRMS (FAB) calcd. for C_6_H_11_N_3_OSNa (M + Na)^+^ *m*/*e* 196.0514, found *m*/*e* 196.0515.

#### 4.1.5. Synthesis of 4-Azidobutane-1-sulfinyl chloride (**4**) 

To a solution of thioacetate **3** (13.3 g, 76.84 mmol) in methylene chloride (67 mL) at −20 °C, acetic anhydride (7.3 mL, 7.84 mmol) and sulfuryl chloride (12.4 mL, 153.67 mmol) were added. The resulting mixture was stirred for 1 h at −5 °C, then the solvent was evaporated and the residue was dried under vacuum to give 14.0 g (76.82 mmol, quantitative yield) of **4** as a black low-melting point solid. The crude sulfinyl chloride, which was kept under argon, was used in the following reaction without further purification, in order to preparate sulfinate esters [33,35]. Hydrogen-1 NMR (300 MHz, CDCl_3_) *δ* 3.44–3.36 (m, 4H), 2.07–2.00 (m, 2H), 1.83–1.73 (m, 2H) ppm (Figure S9).

#### 4.1.6. Synthesis of (*S*)-(1,2:5,6-Di-O-Isopropylidene-α-d-glucofuranosyl) 4-Azidobutanesulfinate (**5-(*S*)**) 

To a solution of 1,2:5,6-di-*O*-isopropylidene-α-d-glucofuranosyl (DAGOH) (1.1 g, 4.30 mmol) and DIPEA (2.3 mL, 13.19 mmol) in anhydrous toluene (20 mL), cooled to −78 °C and under an argon atmosphere, 4-azidobutane-1-sulfinyl chloride **4** (1.6 g, 8.60 mmol) was added, while the reaction mixture was vigorously stirred. An hour later, the reaction mixture was treated with 1 M HCl aqueous solution and extracted with CH_2_Cl_2_. Saturated NaHCO_3_ aqueous solution and brine were used to successively wash the combined organic layers. Then, the product was dried over anhydrous Na_2_SO_4_ and evaporated to obtain *S* sulfinate as the major diastereomer with a 94% diastereomeric excess. The crude was purified by column chromatography (hexane/2-propanol 20:1) to give 1.5 g (3.74 mmol, 87% yield) of diastereomerically pure **5-(*S*)** as a yellow oil [33,35]. *R_f_ =* 0.40 (hexane/2-propanol, 15:1); [α]_D_ = −55.20 (*c* = 1.2, CHCl_3_); ^1^H NMR (500 MHz, CDCl_3_) δ 5.91 (d, 1H, *J* = 3.7 Hz), 4.75 (d, 1H, *J* = 2.7 Hz), 4.61 (d, 1H, *J* = 3.7 Hz), 4.31–4.24 (m, 2H), 4.11–4.08 (m, 1H), 4.01 (dd, 1H, *J* = 5.1 and 8.5 Hz), 3.35 (td, 2H, *J* = 1.6 and 6.4 Hz), 2.89–2.76 (m, 2H), 1.86–1.79 (m, 2H), 1.75–1.70 (m, 2H), 1.51 (s, 3H), 1.43 (s, 3H), 1.35 (s, 3H), 1.32(s, 3H) ppm (Figure S10); ^13^C NMR (125 MHz, CDCl_3_) δ 112.6, 109.5, 105.1, 83.7, 80.5, 79.5, 72.5, 67.0, 56.7, 51.0, 28.2, 26.9, 26.8, 26.4, 25.4, 18.9 ppm (Figure S11); HRMS (FAB) calcd. for C_16_H_28_N_3_O_7_S (M+H)^+^ *m*/*e* 406.1648, found *m*/*e* 406.1649.

#### 4.1.7. Synthesis of (*R*)-(-)-1-Azido-4-(methylsulfinyl)-butane (**6-(*R*)**) 

Methyl magnesium bromide 1.4 M (0.4 mL, 0.56 mmol) was added to a solution of sulfinate **5-(*S*)** (345 mg, 0.85 mmol) in anhydrous toluene (15 mL), at 0 °C. Saturated NH_4_Cl aqueous solution was added after stirring for 1 h at 0 °C. CH_2_Cl_2_ was used to extract the aqueous layer and then the organic layers obtained were combined, dried on anhydrous Na_2_SO_4_ and concentrated. The crude product was purified by column chromatography (EtOAc/MeOH 12:1) to give 127 mg of **6-(*R*)** (0.79 mmol, 93% yield) as a colourless liquid [33,35]. *R*_f_ = 0.31 (EtOAc/MeOH, 9:1); [α]_D_ = −81.53 (*c* = 1.1, CHCl_3_); ^1^H NMR (500 MHz, CDCl_3_) *δ* 3.36 (td, 2H, *J* = 2.2 and 6.6 Hz), 2.78–2.66 (m, 2H), 2.58 (s, 3H), 1.928–1.859 (m, 2H), 1.837–1.71 (m, 2H) ppm (Figure S12); ^13^C NMR (125 MHz, CDCl_3_) *δ* 54.1, 51.0, 38.8, 28.2, 20.2 ppm (Figure S13); HRMS (FAB) *m*/*e* calcd. for C_5_H_11_N_3_NaOS (M+Na)^+^: 184.0514, found: 184.0515.

#### 4.1.8. Synthesis of (*R*)-(-)-1-Isothiocyanato-4-(methylsulfinyl)-butane (***R*-Sulforaphane**) 

Triphenylphosfine (343 mg, 1.31 mmol) was added to a solution of azide **6-(*R*)** (140 mg, 0.69 mmol) in Et_2_O (5 mL), followed by refluxing for an hour. Carbon disulfide (3 mL) was added after removing the solvent at vacuum, leaving the reaction reflux for 3 h. Finally, the solvent was removed under vacuum and the crude product was purified by column chromatography (EtOAc/MeOH 9:1) to give 149 mg of **(*R*)-sulforaphane** (0.68 mmol, 98% yield) as a colourless liquid [33,35]. *R*_f_ = 0.15 (EtOAc/MeOH, 9:1); [α]_D_ = −80.81 (*c* = 1.0, CHCl_3_); ^1^H NMR (500 MHz, CDCl_3_) *δ* 3.59 (t, 2H, *J* = 5.6 Hz), 2.79–2.68 (m, 2H), 2.60 (s, 3H), 1.98–1.83 (m, 4H) ppm (Figure S14); ^13^C NMR (125 MHz, CDCl_3_) *δ* 53.7, 44.8, 38.9, 29.2, 20.3 ppm (Figure S15); HRMS (FAB) *m*/*e* calcd. for C_6_H_12_NOS_2_ (M+H)^+^: 178.0360, found: 178.0358.

### 4.2. Animals

Swiss female mice (8–10 weeks of age) were obtained from the Animal Production Center of the University of Seville (Seville, Spain), placed in cages (6 mice/cage), kept under constant conditions of temperature (20–25 °C) and humidity (40–60%), with a 12 h light/dark cycle, and fed a standard rodent diet (Panlab A04, Panlab, Seville, Spain) and water ad libitum throughout the experiment. All experiments were carried out in the Faculty of Pharmacy (University of Seville, Seville, Spain) in accordance with the recommendations of the European Union (Directive of the European Counsel 2012/707/EU) regarding animal experimentation and following a protocol approved by the Animal Ethics Committee of the University of Seville (CEEA-US 2018-11/2 and CEEA-US 2019-6) and by the Consejeria de Agricultura, Pesca y Desarrollo (Junta de Andalucía, 23/07/2018/119), according to the RD 53/1 February 2013.

### 4.3. Isolation and In Vitro Culture of Murine Peritoneal Macrophages and Spleen Cells

The method used was as described by Aparicio-Soto et al. [74]. After a week of acclimatization of the animals, 1 mL of sodium thioglycolate solution (3.8% *w*/*v*) (BD Difco, Le Pont de Claix, France) was administered to the mice, leaving food and drink ad libitum. After 72 h, the mice were sacrificed by CO_2_ exposure and the peritoneal cavities were washed with cold sterile phosphate-buffered saline solution (PBS) to obtain cellular exudates. Once the cells were centrifuged, they were resuspended in RPMI1640 culture medium supplemented with 10% heat-inactivated fetal calf serum (FCS) in the presence of 100 mg/mL streptomycin and 100 U/mL penicillin and seeded onto plates (1 × 10^6^ cells/mL) for 2 h at 37 °C in a humidified atmosphere of 5% CO_2_. After 2 h, non-adherent cells were eliminated by washing with PBS, and fresh RPMI 1640 without FCS containing 6.25 or 12.5 µM of *(R)*-SFN was added. Thirty minutes later, the cells were stimulated with 5 µg/mL of LPS from Escherichia coli (Sigma-Aldrich, St. Louis, MO, USA) for 18 h in the presence or absence of the study compound. Ultimately, the supernatants were collected.

The spleens were extracted, crushed and filtered using a nylon cell filter (70 µm) (BD^®^ Biosciences, Franklin Lakes, NJ, USA) to prepare a cell suspension. Splenocytes were cultivated in supplemented RPMI1640 culture medium (10% FCS, 100 mg/mL streptomycin and 100 U/mL penicillin, 2 mM L-glutamine, 50 µM 2-mercaptoethanol and 1 mM sodium pyruvate). The cell suspension was centrifuged and resuspended in red cell lysis buffer to remove erythrocytes (BD^®^ Biosciences, Franklin Lakes, NJ, USA). Subsequently, the cells were washed with PBS. Finally, the cells were centrifuged and resuspended in RPMI 1640 supplemented medium to obtain a cell suspension of 3 × 10^6^ cells/mL and were cultured in 24-well plates at 37 °C in humidified air with 5% CO_2_ for 24 h in the presence or absence of (*R*)-SFN (12.5 or 6.25 µM) and LPS (5 µg/mL). Once the culture time had passed, the cells were centrifuged and the cell pellets and supernatants were collected and stored at −80 °C until the ELISA assays and histone extractions were conducted.

### 4.4. Cell Viability Assay

Cellular viability was assessed by the SRB assay [75]. Cells were cultured at 1 × 10^5^ cells/well and incubated in the presence or absence of (*R*)-SFN (200 µM–1.6 µM) for 18 h at 37 °C in a 5% CO_2_ humidified atmosphere. After the incubation time, adherent cells were fixed in situ with 50 µL of cold trichloroacetic acid (Sigma-Aldrich^®^, St. Louis, MO, USA) solution (50% *w*/*v*) and incubated for 1 h at 4 °C. The supernatant was discarded, and the plates were washed with deionized water 5 times and allowed to air dry. Then, 100 µL of the SRB (Sigma-Aldrich^®^, St. Louis, MO, USA) solution (0.4% *w*/*v*) was added to each well, followed by incubation in the dark and at room temperature for 30 min. The supernatant was discarded and washed 5 times with 1% acetic acid (Panreac^®^, Barcelona, Spain), allowing it to air dry. Finally, 100 µL per well of 10 mmol/L Tris base (pH 10.5) (Sigma-Aldrich^®^, St. Louis, MO, USA) was added. The cell viability was quantified by optical density at 492 nm using a multi-well plate reader spectrophotometer (BioTek^®^, Bad Friedrichshall, Germany). Cell survival was measured as a percentage of absorbance by comparing treated cells with control cells (untreated, 100% cell survival).

In each experiment, the viability was always ≥80%. *(R)*-SFN stock solutions were prepared in DMSO (Sigma-Aldrich^®^, St. Louis, MO, USA) and diluted to achieve the desired concentrations in the culture medium. The DMSO concentration in the culture medium was ≤1% in all experiments, which had no significant effects on cells.

### 4.5. Measurement of Nitrite Production

To a 96-well plate together with a standard curve of sodium nitrite, 100 µL of cell supernatants were transferred and subsequently mixed with Griess reagent (Sigma-Aldrich^®^, St. Louis, MO, USA). The plate was then incubated at room temperature for 15 min. Finally, the absorbance at 540 nm was measured using an ELISA reader (BioTek^®^, Bad Friedrichshall, Germany). The amount of nitrite was determined as the NO generation index and obtained by extrapolating a standard curve with sodium nitrite. The results were expressed as the percentage of nitrite production compared to DMSO-LPS cells (stimulated untreated cells).

### 4.6. Detection of DCFDA Cell-Reactive Oxygen Species

The quantification of the intracellular ROS concentration was performed using a DCFDA assay kit (Abcam^®^, Cambridge, UK), according to the manufacturer’s instructions. For this, 2.5 × 10^4^ cells/well were seeded in a black 96-well plate pretreated with (*R*)-SFN (12.5 and 6.25 µM). Thirty minutes later, both treated and untreated cells were stimulated with LPS. Next, DCFDA (25 µM) was added to each of the wells and the cells were incubated at 37 °C for 45 min. Excitation and emission wavelengths (485 and 535 nm, respectively) were read using a fluorescence microplate reader (SynergyTM HTX BioTek^®^, Bad Friedrichshall, Germany). H_2_O_2_ (Sigma-Aldrich^®^, Cambridge, UK) was used as a positive pro-oxidant control (100% intracellular ROS production).

### 4.7. Determination of Pro-Inflammatory Cytokines

The supernatants obtained from the murine peritoneal macrophage and murine splenocyte culture were collected 18 or 24 h after LPS stimulation. Subsequently, they were processed by ELISA kits to determine levels of IL-1β (BD OptEIA^®^, San Jose, CA, USA), IL-6 (Diaclone^®^, Besacon Cedex, France), IL-17 and TNF-α (Peprotech^®^, London, UK), according to the manufacturer’s instructions. Results were expressed in pg/mL.

### 4.8. Histone Extraction

The method used was as described by Hajji et al., based on acid extraction of histones, with slight modifications [76]. Splenocytes were washed twice with cold PBS. Cells were centrifuged 900× *g* for 5 min at 4 °C. Then, the pellet was resuspended with 1 mL of Lysis buffer (10 mM Tris pH 6.5, 50 mM sodium bisulfite, 10 nM MgCl_2_, 8.6% sucrose, 1% Triton X-100), incubated on ice for 15 min. Once the time had elapsed, it was centrifuged at 3500 rpm for 10 min at 4 °C, after which the supernatant was discarded and 1 mL of Tris–EDTA buffer (10 mM Tris pH 7.4 and 12 mM EDTA) was added to the sample. The pellet was resuspended with sulfuric acid 0.2 M. Subsequently, with at least 1h of incubation, the samples were centrifuged at 15,000 rpm for 1 h at 4 °C. The supernatants were collected and incubated with acetone overnight at −20 °C. Finally, the samples were centrifuged at 15,000 rpm for 10 min at 4 °C, the supernatant was discarded and the pellet was resuspended in H_2_O milli-Q, after which the protein content was determined following the method described by Bradford et al. [77].

### 4.9. Immunoblotting Detection

To isolate the proteins, after incubation, cells were rinsed, scraped and collected in ice-cold PBS containing a cocktail of phosphatase and protease inhibitors, as described by Sánchez-Hidalgo et al. [78]. To quantify protein concentration in each of the samples, the reagent protein assay (Bio-Rad^®^, Hercules, CA, USA) was used, using a standard such as γ-globulin, following the method described by Bradford et al. [77]. Subsequently, the samples were aliquoted so that they contained the same amount of protein (15 µg). These were separated on 10% or 15% acrylamide gels (depending on the proteins to be quantified) by sodium dodecyl sulfate–polyacrylamide gel electrophoresis. Then, the proteins were electrophoretically transferred to a nitrocellulose membrane and incubated with specific primary antibodies: rabbit anti-COX-2, rabbit anti-iNOS, rabbit anti-Nrf-2, rabbit anti-pSTAT3, rabbit anti-pJAK2, rabbit anti-pJNK, rabbit anti-JNK, rabbit anti-pp38, rabbit anti-p38, rabbit anti-pERK 1/2, mouse anti-ERK 1/2, rabbit anti-NLRP3, rabbit anti-ASC, rabbit anti-caspase-1, rabbit anti-H3K18ac, rabbit anti-H3K27me3, rabbit anti-H3K9me3, mouse anti-H3 (Cell Signaling Technology^®^, Danvers, MA, USA) (1:1000), rabbit anti-mPGES1, rabbit anti-IL18 (Abcam^®^, Cambridge, UK) (1:1000), rabbit anti-caspase-11 (Novus Biologicals^®^, Littleton, CO, USA) (1:500) and rabbit anti-HO-1 (Enzo^®^, Madrid, Spain) (1:1000), overnight at 4 °C. After rinsing, the membranes were incubated with a horseradish peroxidase-labeled secondary antibody anti-rabbit (Cell Signaling Technology^®^, Danvers, MA, USA) (1:2000) or anti-mouse (Dako^®^, Atlanta, GA, USA) (1:2000) containing blocking solution for 1–2 h at room temperature. To prove equal loading, the blots were analyzed for β-actin expression using a mouse anti-β-actin antibody (Abcam^®^, Cambridge, UK) (1:10,000). Immunodetection was performed using an enhanced chemiluminescence light detection kit (Pierce^®^, Rockford, IL, USA).

Immune signals were captured using the Amersham^TM^ Imager 600 from GE Healthcare^®^ (Buckinghamshire, UK), and densitometric data were studied after normalization to the housekeeping loading control. The signals were analyzed and quantified by Image Processing and Analysis in Java (Image J^®^, Bethesda, MD, USA) and expressed in relation to the DMSO-LPS treated cells.

### 4.10. Statistical Evaluation

Data were evaluated with GraphPad Prism version 5.01 software (San Diego, CA, USA). All values in the figures and text are expressed as arithmetic means ± SEMs. One-way analysis of variance (ANOVA) was used to evaluate the statistical significance of any difference in each parameter between groups, and after that the Tukey–Kramer multiple comparison test was used as a post hoc test. *p*-values < 0.05 were considered statistically significant. Experiments were carried out in triplicate, and specifically, in the densitometry assays, the figures shown are representative of at least six different experiments performed on different days.

## 5. Conclusions

Taken together, these findings demonstrate, for the first time, that (*R*)-SFN was able to modulate the inflammatory response and oxidative stress induced by LPS stimulation in murine peritoneal macrophages, reducing pro-inflammatory enzyme expression (iNOS, COX-2 and mPGES-1) and cytokine production (IL-1β, IL-6, IL-17, IL-18 and TNF-α) via inhibition of MAPK, JAK2/STAT-3 and canonical and non-canonical inflammasome signaling pathways. Additionally, this natural isothiocyanate could reduce NO and ROS levels and up-regulate the Nrf-2/HO-1 axis. Lastly, in spleen cells activated by LPS, (*R*)-SFN modulated epigenetic changes through histone methylation (H3K9me3) and deacetylation (H3K18ac). Consequently, (*R*)-SFN could be a new epinutraceutical compound useful for the management of several immunoinflammatory diseases.

## Data Availability

Data are contained within the article.

## Supplementary Materials

The following supporting information can be downloaded at: https://www.mdpi.com/article/10.3390/ph15080966/s1, Figure S1: Sulforaphane, thioether (I) and sulfone (II) derivatives; Figure S2: Scheme of DAG methodology; Figure S3: ^1^H-NMR of 4-Azidobutan-1-ol (**1**); Figure S4: ^13^C-NMR of 4-Azidobutan-1-ol (**1**); Figure S5: ^1^H-NMR of 4-Azidobutyl methanesulfonate (**2**); Figure S6: ^13^C-NMR of 4-Azidobutyl methanesulfonate (**2**); Figure S7: ^1^H-NMR of 4-Azidobutyl-1-thioacetate (**3**); Figure S8: ^13^C-NMR of 4-Azidobutyl-1-thioacetate (**3**); Figure S9: ^1^H-NMR of 4-Azidobutane-1-sulfinyl chloride (**4**); Figure S10: ^1^H-NMR of (*S*)-(1,2:5,6-Di-O-isopropylidene-α-D-glucofuranosyl) 4-azidobutanesulfinate (**5-(*S*)**); Figure S11: ^13^C-NMR of (*S*)-(1,2:5,6-Di-O-isopropylidene-α-D-glucofuranosyl) 4-azidobutanesulfinate (**5-(*S*)**); Figure S12: ^1^H-NMR of (*R*)-(-)-1-Azido-4-(methylsulfinyl)-butane (**6-(*R*)**); Figure S13: ^13^C-NMR of (*R*)-(-)-1-Azido-4-(methylsulfinyl)-butane (**6-(*R*)**); Figure S14: ^1^H-NMR of (*R*)-(-)-1-Isothiocyanato-4-(butylsulfinyl)-butane (***R*-sulforaphane**); Figure S15: ^13^C-NMR of (*R*)-(-)-1-Isothiocyanato-4-(butylsulfinyl)-butane (***R*-sulforaphane**).

## References

[1] Mangla B., Javed S., Sultan M.H., Kumar P., Kohli K., Najmi A., Alhazmi H.A., Al Bratty M., Ahsan W. (2021). Sulforaphane: A review of its therapeutic potentials, advances in its nanodelivery, recent patents, and clinical trials. Phytother. Res..

[2] Rose P. (2000). 7-Methylsulfinylheptyl and 8-methylsulfinyloctyl isothiocyanates from watercress are potent inducers of phase II enzymes. Carcinogenesis.

[3] Zhang Y., Talalay P., Cho C.G., Posner G.H. (1992). A major inducer of anticarcinogenic protective enzymes from broccoli: Isolation and elucidation of structure. Proc. Natl. Acad. Sci. USA.

[4] Juge N., Mithen R.F., Traka M. (2007). Molecular basis for chemoprevention by sulforaphane: A comprehensive review. Cell. Mol. Life Sci..

[5] Vergara F., Wenzler M., Hansen B.G., Kliebenstein D.J., Halkier B.A., Gershenzon J., Schneider B. (2008). Determination of the absolute configuration of the glucosinolate methyl sulfoxide group reveals a stereospecific biosynthesis of the side chain. Phytochemistry.

[6] Houghton C.A., Fassett R.G., Coombes J.S. (2016). Sulforaphane and Other Nutrigenomic Nrf2 Activators: Can the Clinician’s Expectation Be Matched by the Reality?. Oxidative Med. Cell. Longev..

[7] Lee J.H., Moon M.H., Jeong J.K., Park Y.G., Lee Y.J., Seol J.W., Park S.Y. (2012). Sulforaphane induced adipolysis via hormone sensitive lipase activation, regulated by AMPK signaling pathway. Biochem. Biophys. Res. Commun..

[8] Johansson N.L., Pavia C.S., Jen W.C. (2008). Growth inhibition of a spectrum of bacterial and fungal pathogens by sulforaphane, an isothiocyanate product found in broccoli and other cruciferous vegetables. Planta Med..

[9] Amjad A.I., Parikh R.A., Appleman L.J., Hahm E.-R., Singh K., Singh S.V. (2015). Broccoli-Derived Sulforaphane and Chemoprevention of Prostate Cancer: From Bench to Bedside. Curr. Pharmacol. Rep..

[10] Giacoppo S., Galuppo M., Montaut S., Iori R., Rollin P., Bramanti P., Mazzon E. (2015). An overview on neuroprotective effects of isothiocyanates for the treatment of neurodegenerative diseases. Fitoterapia.

[11] Bai Y., Wang X., Zhao S., Ma C., Cui J., Zheng Y. (2015). Sulforaphane Protects against Cardiovascular Disease via Nrf2 Activation. Oxidative Med. Cell. Longev..

[12] Lauterbach M.A., Hanke J.E., Serefidou M., Mangan M.S.J., Kolbe C.C., Hess T., Rothe M., Kaiser R., Hoss F., Gehlen J. (2019). Toll-like Receptor Signaling Rewires Macrophage Metabolism and Promotes Histone Acetylation via ATP-Citrate Lyase. Immunity.

[13] Yi Y.S. (2017). Caspase-11 non-canonical inflammasome: A critical sensor of intracellular lipopolysaccharide in macrophage-mediated inflammatory responses. Immunology.

[14] Jeong Y.H., Oh Y.C., Cho W.K., Yim N.H., Ma J.Y. (2019). Hoveniae semen seu fructus ethanol extract exhibits anti-inflammatory activity via MAPK, AP-1, and STAT signaling pathways in LPS-stimulated RAW 264.7 and mouse peritoneal macrophages. Mediat. Inflamm..

[15] Li R., Hong P., Zheng X. (2019). β-carotene attenuates lipopolysaccharide-induced inflammation via inhibition of the NF-κB, JAK2/STAT3 and JNK/p38 MAPK signaling pathways in macrophages. Anim. Sci. J..

[16] Huang Y.-P., Chen D.-R., Lin W.-J., Lin Y.-H., Chen J.-Y., Kuo Y.-H., Chung J.-G., Hsia T.-C., Hsieh W.-T. (2021). Ergosta-7,9(11),22-trien-3β-ol Attenuates Inflammatory Responses via Inhibiting MAPK/AP-1 Induced IL-6/JAK/STAT Pathways and Activating Nrf2/HO-1 Signaling in LPS-Stimulated Macrophage-like Cells. Antioxidants.

[17] Zhang Y., Tang L. (2007). Discovery and development of sulforaphane as a cancer chemopreventive phytochemical. Acta Pharmacol. Sin..

[18] Abdull Razis A.F., Iori R., Ioannides C. (2011). The natural chemopreventive phytochemical R-sulforaphane is a far more potent inducer of the carcinogen-detoxifying enzyme systems in rat liver and lung than the S-isomer. Int. J. Cancer.

[19] Abdull Razis A.F., Bagatta M., De Nicola G.R., Iori R., Ioannides C. (2011). Induction of epoxide hydrolase and glucuronosyl transferase by isothiocyanates and intact glucosinolates in precision-cut rat liver slices: Importance of side-chain substituent and chirality. Arch. Toxicol..

[20] Liang J., Jahraus B., Balta E., Ziegler J.D., Hübner K., Blank N., Niesler B., Wabnitz G.H., Samstag Y. (2018). Sulforaphane inhibits inflammatory responses of primary human T-cells by increasing ROS and depleting glutathione. Front. Immunol..

[21] Rakariyatham K., Wu X., Tang Z., Han Y., Wang Q., Xiao H. (2018). Synergism between luteolin and sulforaphane in anti-inflammation. Food Funct..

[22] Ruhee R.T., Ma S., Suzuki K. (2019). Sulforaphane protects cells against lipopolysaccharide-stimulated inflammation in murine macrophages. Antioxidants.

[23] Ranaweera S.S., Dissanayake C.Y., Natraj P., Lee Y.J., Han C.H. (2020). Anti-inflammatory effect of sulforaphane on LPS-stimulated RAW 264.7 cells and ob/ob mice. J. Vet. Sci..

[24] Qi T., Xu F., Yan X., Li S., Li H. (2016). Sulforaphane exerts anti-inflammatory effects against lipopolysaccharide-induced acute lung injury in mice through the Nrf2/ARE pathway. Int. J. Mol. Med..

[25] Mohanty S., Pal A., Konkimalla V.B., Sudam Chandra S.I. (2018). Anti-inflammatory and anti-granuloma activity of sulforaphane, a naturally occurring isothiocyanate from broccoli (Brassica oleracea). Asian J. Pharm. Clin. Res..

[26] Li B., Cui W., Liu J., Li R., Liu Q., Xie X.H., Ge X.L., Zhang J., Song X.J., Wang Y. (2013). Sulforaphane ameliorates the development of experimental autoimmune encephalomyelitis by antagonizing oxidative stress and Th17-related inflammation in mice. Exp. Neurol..

[27] Zeng X., Liu X., Bao H. (2021). Sulforaphane suppresses lipopolysaccharide- and Pam3CysSerLys4-mediated inflammation in chronic obstructive pulmonary disease via toll-like receptors. FEBS Openbio.

[28] Fernandez I., Khiar N., Llera J.M., Alcudia F. (1992). Asymmetric synthesis of alkane- and arenesulfinates of diacetone-D-glucose (DAG): An improved and general route to both enantiomerically pure sulfoxides. J. Org. Chem..

[29] Fernández I., Khiar N. (2003). Recent developments in the synthesis and utilization of chiral sulfoxides. Chem. Rev..

[30] Khiar N., Alcudia F., Espartero J.L., Rodríguez L., Fernández I. (2000). Dynamic kinetic resolution of bis-sulfinyl chlorides: A general enantiodivergent synthesis of C2-symmetric bis-sulfinate esters and bis-sulfoxides. J. Am. Chem. Soc..

[31] Balcells D., Ujaque G., Fernández I., Khiar N., Maseras F. (2007). How does the achiral base decide the stereochemical outcome in the dynamic kinetic resolution of sulfinyl chlorides? A computational study. Adv. Synth. Catal..

[32] Khiar el Wahabi N., Fernández Fernández I., Recio Jiménez R. (2013). Sulforaphane-Derived Compounds, Production Method Thereof and the Medical, Food and Cosmetic Use of Same. http://digital.csic.es/handle/10261/92880.

[33] Elhalem E., Recio R., Werner S., Lieder F., Calderón-Montaño J.M., López-Lázaro M., Fernández I., Khiar N. (2014). Sulforaphane homologues: Enantiodivergent synthesis of both enantiomers, activation of the Nrf2 transcription factor and selective cytotoxic activity. Eur. J. Med. Chem..

[34] Recio R., Elhalem E., Benito J.M., Fernández I., Khiar N. (2018). NMR study on the stabilization and chiral discrimination of sulforaphane enantiomers and analogues by cyclodextrins. Carbohydr. Polym..

[35] Khiar N., Werner S., Mallouk S., Lieder F., Alcudia A., Fernández I. (2009). Enantiopure sulforaphane analogues with various substituents at the sulfinyl sulfur: Asymmetric synthesis and biological activities. J. Org. Chem..

[36] Recio R., Vengut-Climent E., Borrego L.G., Khiar N., Fernández I., Atta-ur R. (2017). Biologically Active Isothiocyanates: Protecting Plants and Healing Humans. Studies in Natural Products Chemistry.

[37] Caradonna F., Consiglio O., Luparello C., Gentile C. (2020). Science and healthy meals in the world: Nutritional epigenomics and nutrigenetics of the mediterranean diet. Nutrients.

[38] Yuan F., Chen X., Liu J., Feng W., Cai L., Wu X., Chen S.Y. (2018). Sulforaphane restores acetyl-histone H3 binding to Bcl-2 promoter and prevents apoptosis in ethanol-exposed neural crest cells and mouse embryos. Exp. Neurol..

[39] Su X., Jiang X., Meng L., Dong X., Shen Y., Xin Y. (2018). Anticancer activity of sulforaphane: The epigenetic mechanisms and the Nrf2 signaling pathway. Oxidative Med. Cell. Longev..

[40] Senanayake G.V.K., Banigesh A., Wu L., Lee P., Juurlink B.H.J. (2012). The dietary phase 2 protein inducer sulforaphane can normalize the kidney epigenome and improve blood pressure in hypertensive rats. Am. J. Hypertens..

[41] Dacosta C., Bao Y. (2017). The role of microRNAs in the chemopreventive activity of sulforaphane from cruciferous vegetables. Nutrients.

[42] Kou X., Qi S., Dai W., Luo L., Yin Z. (2011). Arctigenin inhibits lipopolysaccharide-induced iNOS expression in RAW264.7 cells through suppressing JAK-STAT signal pathway. Int. Immunopharmacol..

[43] Yang Y., Wang H., Kouadir M., Song H., Shi F. (2019). Recent advances in the mechanisms of NLRP3 inflammasome activation and its inhibitors. Cell Death Dis..

[44] Mazarakis N., Anderson J., Toh Z.Q., Higgins R.A., Do L.A.H., Luwor R.B., Snibson K.J., Karagiannis T.C., Licciardi P.V. (2021). Examination of novel immunomodulatory effects of l-sulforaphane. Nutrients.

[45] Li L., Maitra U., Singh N., Gan L. (2010). Molecular mechanism underlying LPS-induced generation of reactive oxygen species in macrophages. FASEB J..

[46] Cuevas B., Arroba A.I., de los Reyes C., Gómez-Jaramillo L., González-Montelongo M.C., Zubía E. (2021). Diterpenoids from the brown alga rugulopteryx okamurae and their anti-inflammatory activity. Mar. Drugs.

[47] Tang H., Roy P., Di Q., Ma X., Xiao Y., Wu Z., Quan J., Zhao J., Xiao W., Chen W. (2022). Synthesis compound XCR-7a ameliorates LPS-induced inflammatory response by inhibiting the phosphorylation of c-Fos. Biomed. Pharmacother..

[48] Mitchell J.A., Larkin S., Williams T.J. (1995). Cyclooxygenase-2: Regulation and relevance in inflammation. Biochem. Pharmacol..

[49] Montoya T., Aparicio-Soto M., Castejón M.L., Rosillo M.Á., Sánchez-Hidalgo M., Begines P., Fernández-Bolaños J.G., Alarcón-de-la-Lastra C. (2018). Peracetylated hydroxytyrosol, a new hydroxytyrosol derivate, attenuates LPS-induced inflammatory response in murine peritoneal macrophages via regulation of non-canonical inflammasome, Nrf2/HO1 and JAK/STAT signaling pathways. J. Nutr. Biochem..

[50] Montoya T., Castejón M.L., Sánchez-Hidalgo M., González-Benjumea A., Fernández-Bolaños J.G., Alarcón De-La-Lastra C. (2019). Oleocanthal Modulates LPS-Induced Murine Peritoneal Macrophages Activation via Regulation of Inflammasome, Nrf-2/HO-1, and MAPKs Signaling Pathways. J. Agric. Food Chem..

[51] Vuong L.D., Nguyen Q.N., Truong V.L. (2019). Anti-inflammatory and anti-oxidant effects of combination between sulforaphane and acetaminophen in LPS-stimulated RAW 264.7 macrophage cells. Immunopharmacol. Immunotoxicol..

[52] Kubo E., Chhunchha B., Singh P., Sasaki H., Singh D.P. (2017). Sulforaphane reactivates cellular antioxidant defense by inducing Nrf2/ARE/Prdx6 activity during aging and oxidative stress. Sci. Rep..

[53] Houghton C.A. (2019). Sulforaphane: Its «Coming of Age» as a Clinically Relevant Nutraceutical in the Prevention and Treatment of Chronic Disease. Oxidative Med. Cell. Longev..

[54] Subedi L., Lee J., Yumnam S., Ji E., Kim S. (2019). Anti-Inflammatory Effect of Sulforaphane on LPS-Activated Microglia Potentially through JNK/AP-1/NF-κB Inhibition and Nrf2/HO-1 Activation. Cells.

[55] Montoya T., Alarcón-De-la-lastra C., Castejón M.L., Ortega-Vidal J., Altarejos J., Sánchez-Hidalgo M. (2022). (-)-methyl-oleocanthal, a new oleocanthal metabolite reduces LPS-induced inflammatory and oxidative response: Molecular signaling pathways and histones epigenetic modulation. Antioxidants.

[56] Arthur J.S.C., Ley S.C. (2013). Mitogen-activated protein kinases in innate immunity. Nat. Rev. Immunol..

[57] Deramaudt T.B., Ali M., Vinit S., Bonay M. (2020). Sulforaphane reduces intracellular survival of Staphylococcus aureus in macrophages through inhibition of JNK and p38 MAPK-induced inflammation. Int. J. Mol. Med..

[58] Wang Z., Zhang S., Xiao Y., Zhang W., Wu S., Qin T., Yue Y., Qian W., Li L. (2020). NLRP3 Inflammasome and Inflammatory Diseases. Oxidative Med. Cell. Longev..

[59] Matikainen S., Nyman T.A., Cypryk W. (2020). Function and Regulation of Noncanonical Caspase-4/5/11 Inflammasome. J. Immunol..

[60] Kiser C., Gonul C.P., Olcum M., Genc S. (2021). Inhibitory effects of sulforaphane on NLRP3 inflammasome activation. Mol. Immunol..

[61] Yu C., He Q., Zheng J., Li L.Y., Hou Y.H., Song F.Z. (2017). Sulforaphane improves outcomes and slows cerebral ischemic/reperfusion injury via inhibition of NLRP3 inflammasome activation in rats. Int. Immunopharmacol..

[62] Li S., Yang H., Chen X. (2019). Protective effects of sulforaphane on diabetic retinopathy: Activation of the nrf2 pathway and inhibition of nlrp3 inflammasome formation. Exp. Anim..

[63] Yang G., Yeon S.H., Lee H.E., Kang H.C., Cho Y.Y., Lee H.S., Lee J.Y. (2018). Suppression of NLRP3 inflammasome by oral treatment with sulforaphane alleviates acute gouty inflammation. Rheumatology.

[64] Lee J., Ahn H., Hong E.J., An B.S., Jeung E.B., Lee G.S. (2016). Sulforaphane attenuates activation of NLRP3 and NLRC4 inflammasomes but not AIM2 inflammasome. Cell. Immunol..

[65] Yang G., Lee H.E., Lee J.Y. (2016). A pharmacological inhibitor of NLRP3 inflammasome prevents non-alcoholic fatty liver disease in a mouse model induced by high fat diet. Sci. Rep..

[66] Fu Q., Zhai Z., Wang Y., Xu L., Jia P., Xia P., Liu C., Zhang X., Qin T., Zhang H. (2018). NLRP3 Deficiency Alleviates Severe Acute Pancreatitis and Pancreatitis-Associated Lung Injury in a Mouse Model. Biomed. Res. Int..

[67] Deng Y., Guo S.L., Wei B., Gao X.C., Zhou Y.C., Li J.Q. (2019). Activation of nicotinic acetylcholine α7 receptor attenuates progression of monocrotaline-induced pulmonary hypertension in rats by downregulating the NLRP3 inflammasome. Front. Pharmacol..

[68] Greaney A.J., Maier N.K., Leppla S.H., Moayeri M. (2016). Sulforaphane inhibits multiple inflammasomes through an Nrf2-independent mechanism. J. Leukoc. Biol..

[69] An Y.W., Jhang K.A., Woo S.Y., Kang J.L., Chong Y.H. (2016). Sulforaphane exerts its anti-inflammatory effect against amyloid-β peptide via STAT-1 dephosphorylation and activation of Nrf2/HO-1 cascade in human THP-1 macrophages. Neurobiol. Aging.

[70] Tufekci K.U., Ercan I., Isci K.B., Olcum M., Tastan B., Gonul C.P., Genc K., Genc S. (2021). Sulforaphane inhibits NLRP3 inflammasome activation in microglia through Nrf2-mediated miRNA alteration. Immunol. Lett..

[71] Zhao S., Zhong Y., Fu X., Wang Y., Ye P., Cai J., Liu Y., Sun J., Mei Z., Jiang Y. (2019). H3K4 Methylation Regulates LPS-Induced Proinflammatory Cytokine Expression and Release in Macrophages. Shock.

[72] Liu K.L., Zhu K., Zhang H. (2022). An overview of the development of EED inhibitors to disable the PRC2 function. RSC Med. Chem..

[73] Yi Y.S. (2021). Functional interplay between methyltransferases and inflammasomes in inflammatory responses and diseases. Int. J. Mol. Sci..

[74] Aparicio-Soto M., Sánchez-Fidalgo S., González-Benjumea A., Maya I., Fernández-Bolaños J.G., Alarcón-de-la-Lastra C. (2015). Naturally occurring hydroxytyrosol derivatives: Hydroxytyrosyl acetate and 3,4-dihydroxyphenylglycol modulate inflammatory response in murine peritoneal macrophages. potential utility as new dietary supplements. J. Agric. Food Chem..

[75] Skehan P., Storeng R., Scudiero D., Monks A., McMahon J., Vistica D., Warren J.T., Bokesch H., Kenney S., Boyd M.R. (1990). New Colorimetric Cytotoxicity Assay for Anticancer-Drug Screening. J. Natl. Cancer Inst..

[76] Hajji N., Joseph B. (2010). Epigenetic regulation of cell life and death decisions and deregulation in cancer. Essays Biochem..

[77] Bradford M. (1976). A Rapid and Sensitive Method for the Quantitation of Microgram Quantities of Protein Utilizing the Principle of Protein-Dye Binding. Anal. Biochem..

[78] Sánchez-Hidalgo M., Martín A.R., Villegas I., Alarcón De La Lastra C. (2005). Rosiglitazone, an agonist of peroxisome proliferator-activated receptor gamma, reduces chronic colonic inflammation in rats. Biochem. Pharmacol..

