# Supplementary material for: Immunomodulatory Effects of (R)-Sulforaphane on LPS-Activated Murine Immune Cells: Molecular Signaling Pathways and Epigenetic Changes in Histone Markers
Source: Pharmaceuticals (Basel). 2022 Aug 4;15(8):966. doi: 10.3390/ph15080966 (PMC9414446; doi:10.3390/ph15080966)
Supplement: Supplementary file 1 [file pharmaceuticals-15-00966-s001.zip › pharmaceuticals-1809325-supplementary.pdf]

Supplementary Files

Immunomodulatory Effects of (*R*)-Sulforaphane on LPS-Activated Murine Immune Cells: Molecular Signaling Pathways and Epigenetic Changes in Histone Markers

Manuel Alcarranza[1*], Isabel Villegas[1], Rocío Muñoz-García[1], Rocío Recio[2], Inmaculada Fernández[2], Catalina Alarcón-de-la-Lastra[1*].

1 Department of Pharmacology, Faculty of Pharmacy, Universidad de Sevilla, 41012 Sevilla, Spain; villegas@us.es (I.V.); rmgarcia@us.es (R.M.-G.)

2 Departamento de Química Orgánica y Farmacéutica, Facultad de Farmacia. Universidad de Sevilla, 41012 Sevilla, Spain; rrecioj@us.es (R.R.); inmaff@us.es (I.F.)

Correspondence: malcarranza@us.es (M.A.); calarcon@us.es (C.A.-d.-l.-L.); Tel.: +34-954559877 (C.A.-d.-l.-L.)

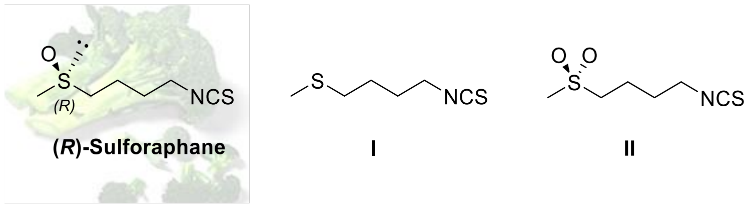

Figure S1. Sulforaphane, thioether (I) and sulfone (II) derivatives.

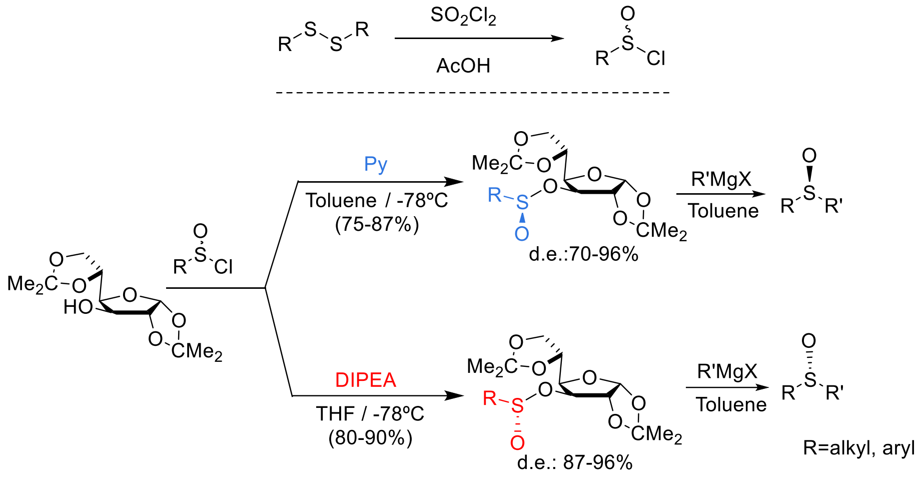

Figure S2. Scheme of DAG-methodology.

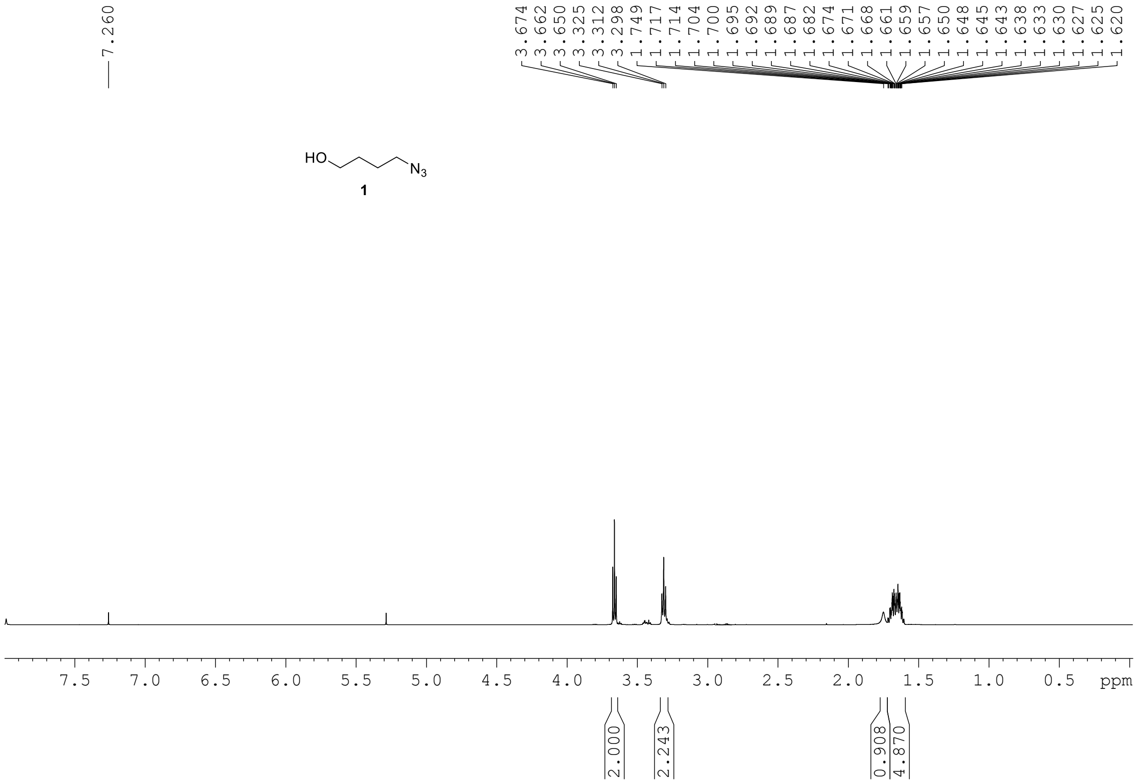

Figure S3. ^{1}H-NMR of 4-Azidobutan-1-ol (**1**)

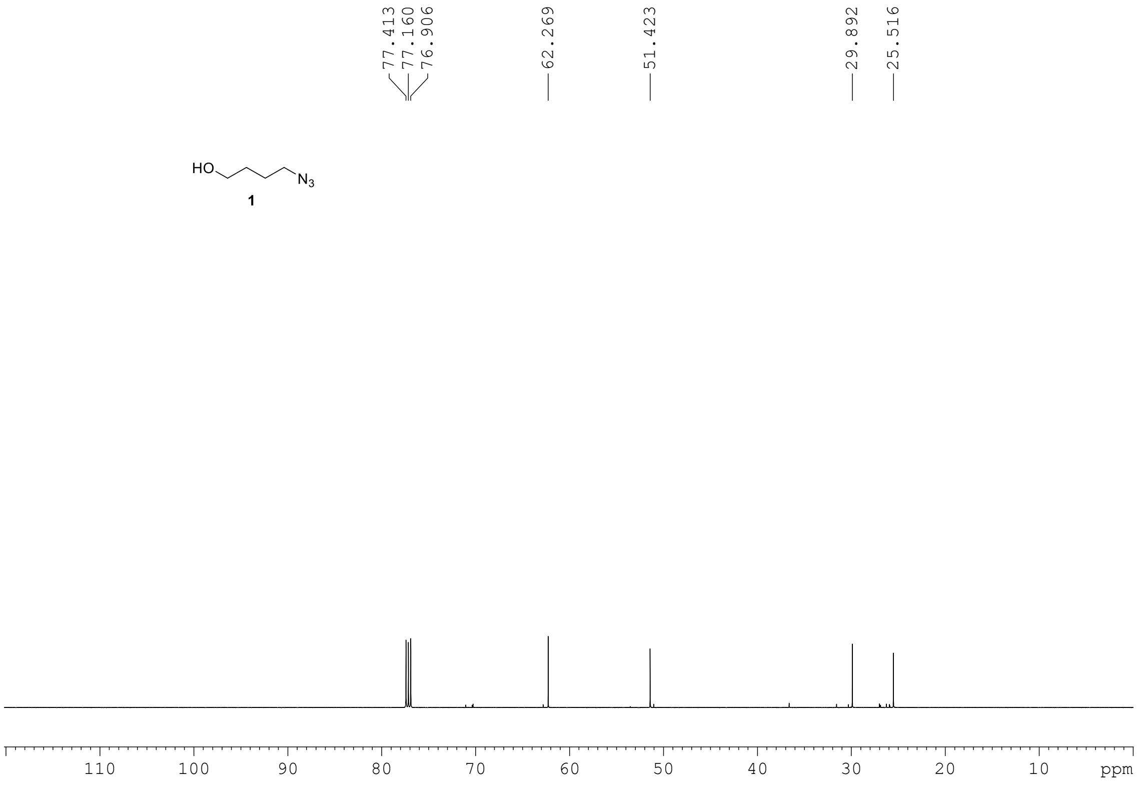

Figure S4. ^{13}C-NMR of 4-Azidobutan-1-ol (**1**)

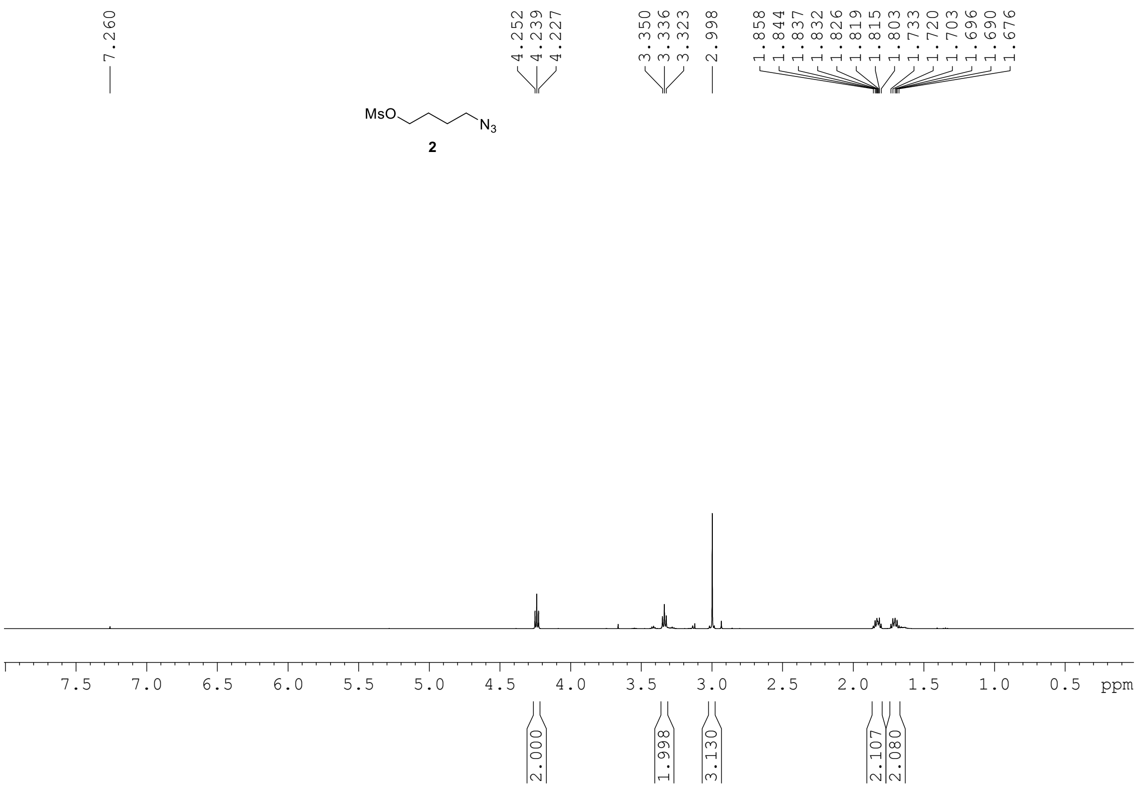

Figure S5. ^{1}H-NMR of 4-Azidobutyl methanesulfonate (**2**)

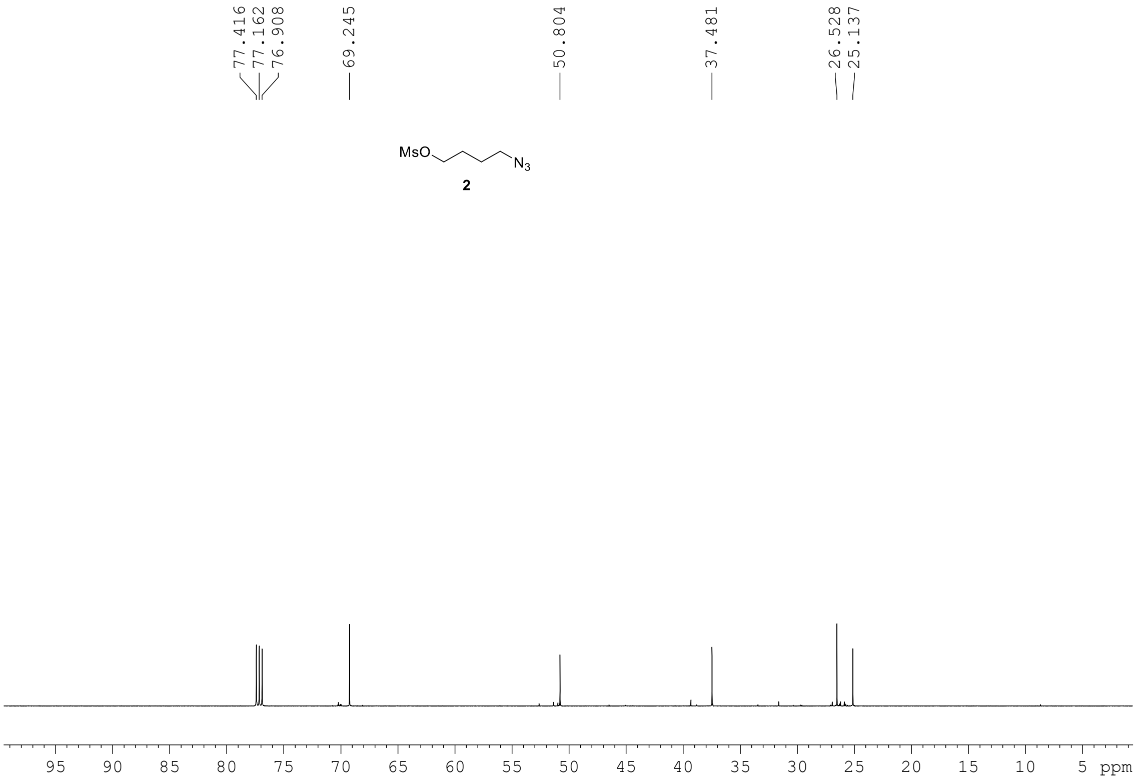

Figure S6. ^{13}C-NMR of 4-Azidobutyl methanesulfonate (**2**)

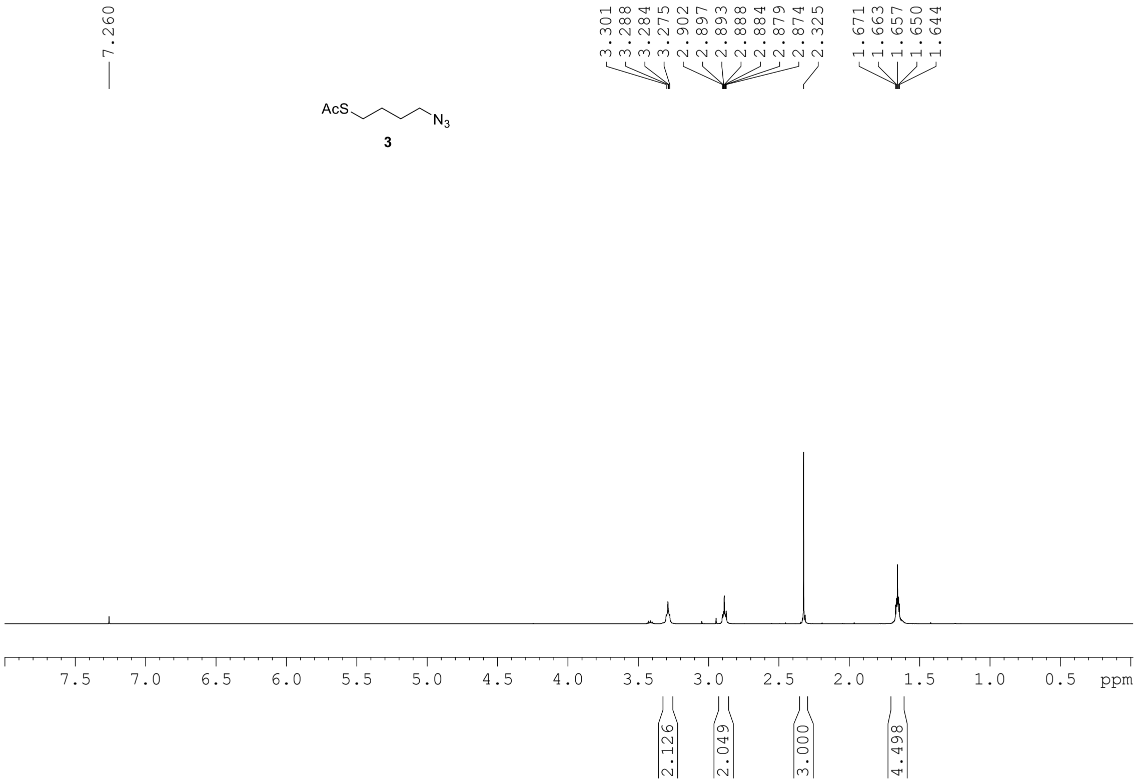

Figure S7. ^{1}H-NMR of 4-Azidobutyl-1-thioacetate (**3**)

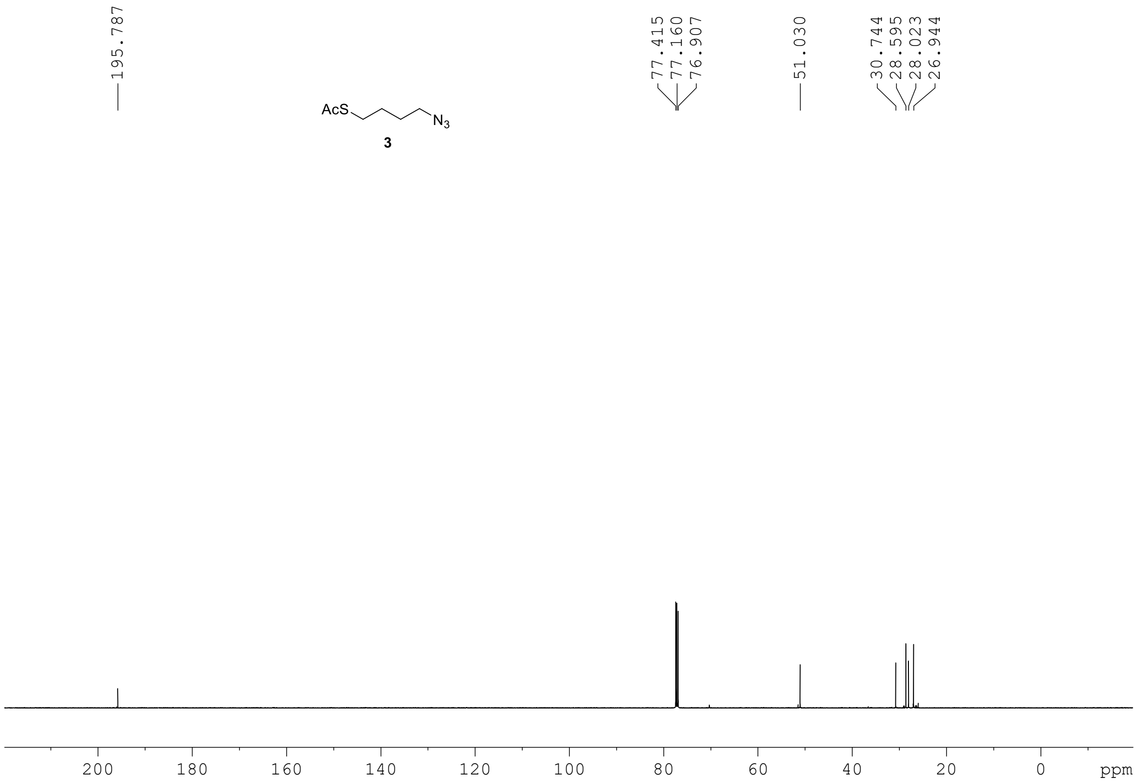

Figure S8. ^{13}C-NMR of 4-Azidobutyl-1-thioacetate (**3**)

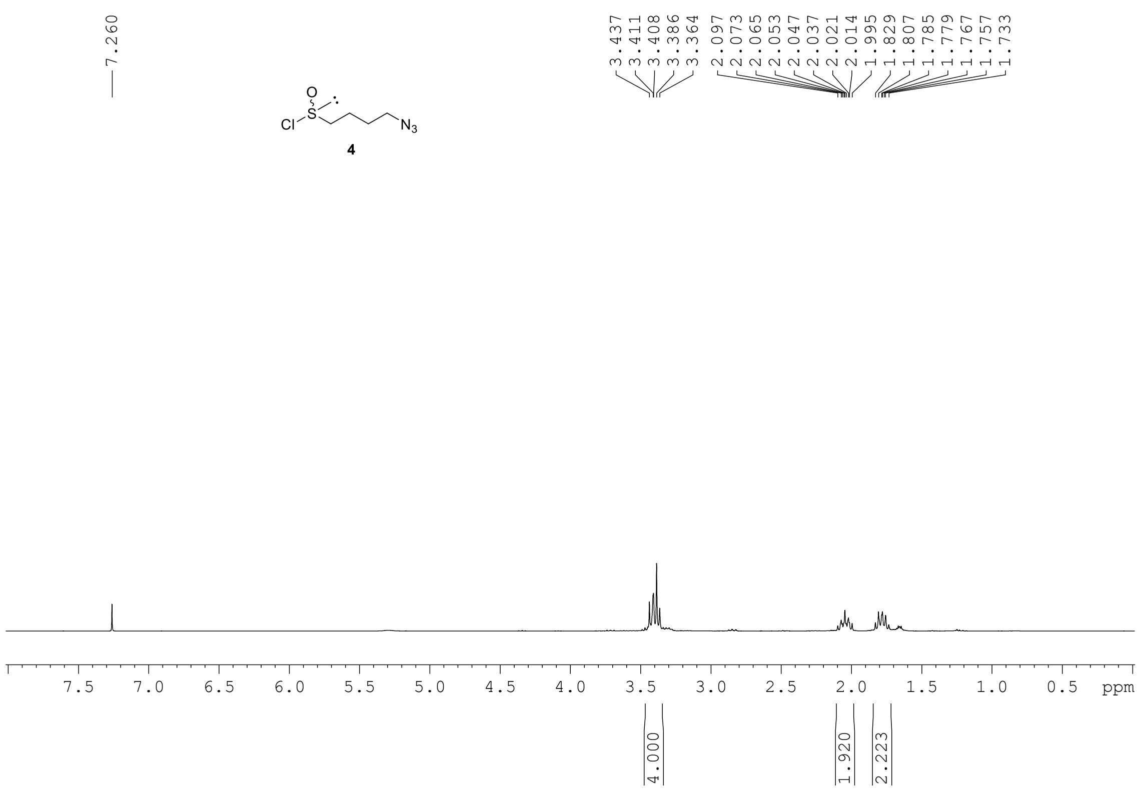

Figure S9. ^{1}H-NMR of 4-Azidobutane-1-sulfinyl chloride (**4**)

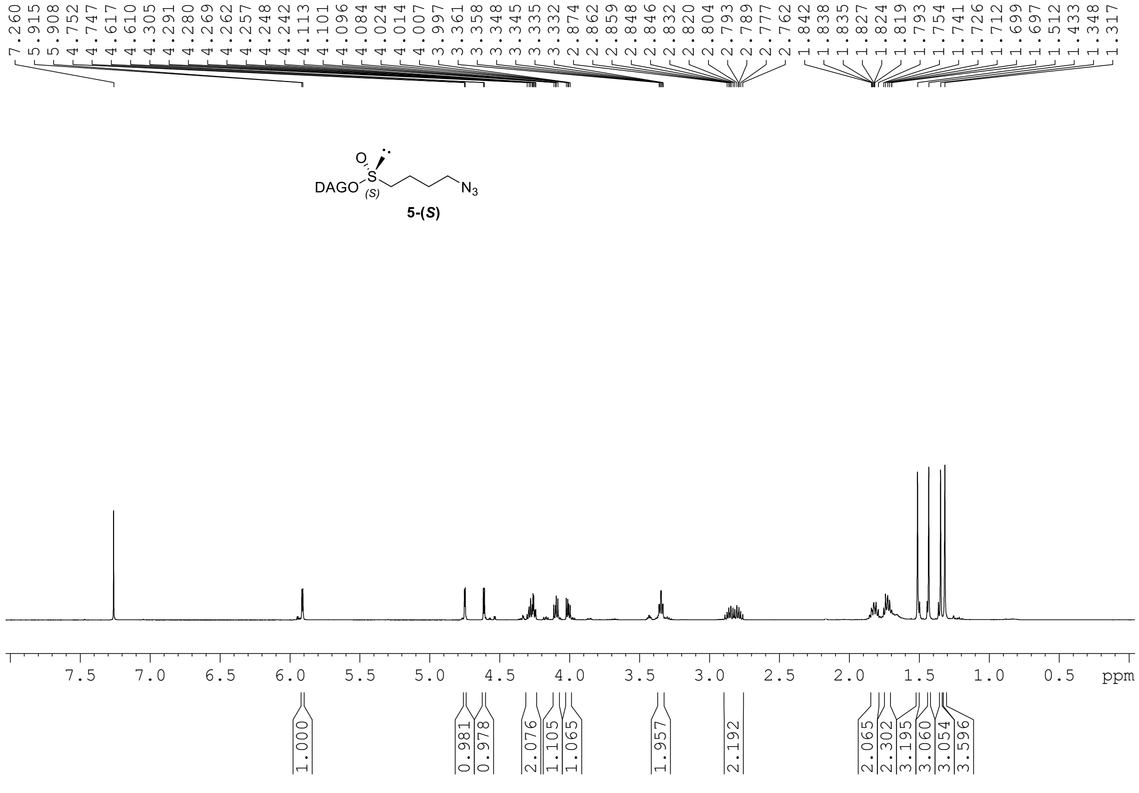

Figure S10. ^{1}H-NMR of (*S*)-(1,2:5,6-Di-O-isopropylidene-α-D-glucofuranosyl) 4-azidobutanesulfinate (**5-(*S*)**)

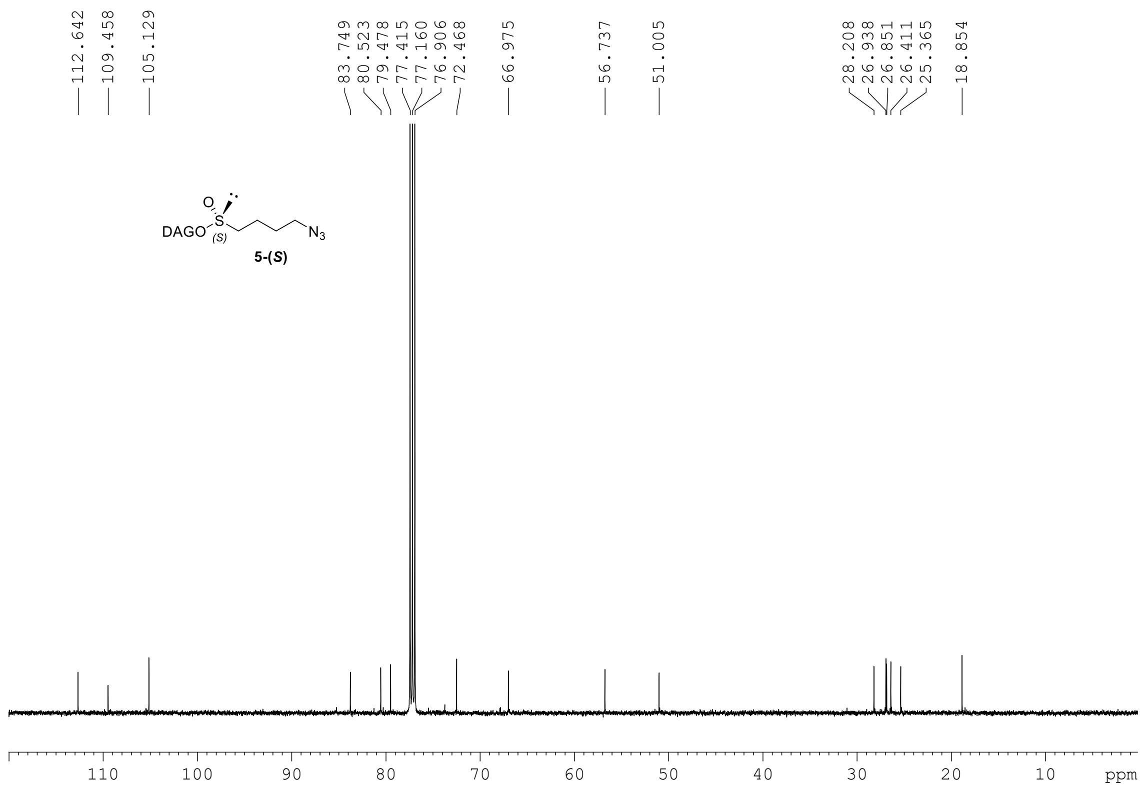

Figure S11. ^{13}C-NMR of (*S*)-(1,2:5,6-Di-O-isopropylidene-α-D-glucofuranosyl) 4-azidobutanesulfinate (**5-(*S*)**)

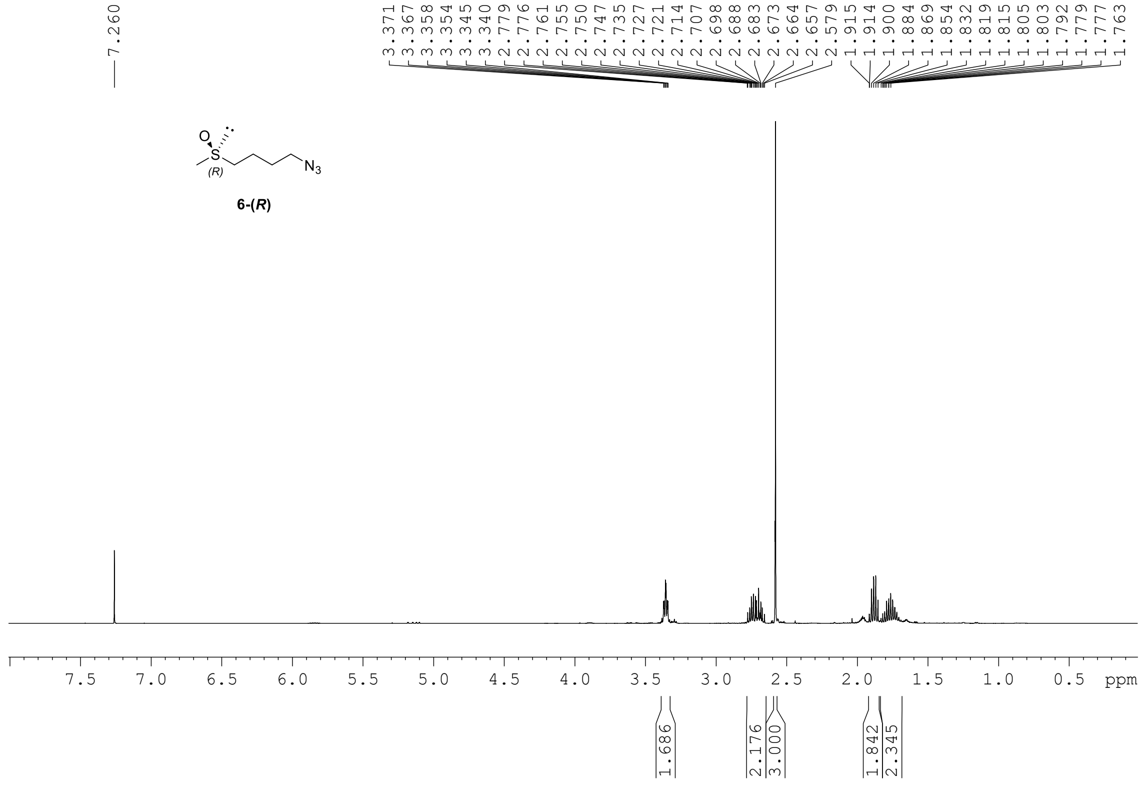

Figure S12. ^{1}H-NMR of (*R*)-(-)-1-Azido-4-(methylsulfinyl)-butane (**6-(*R*)**)

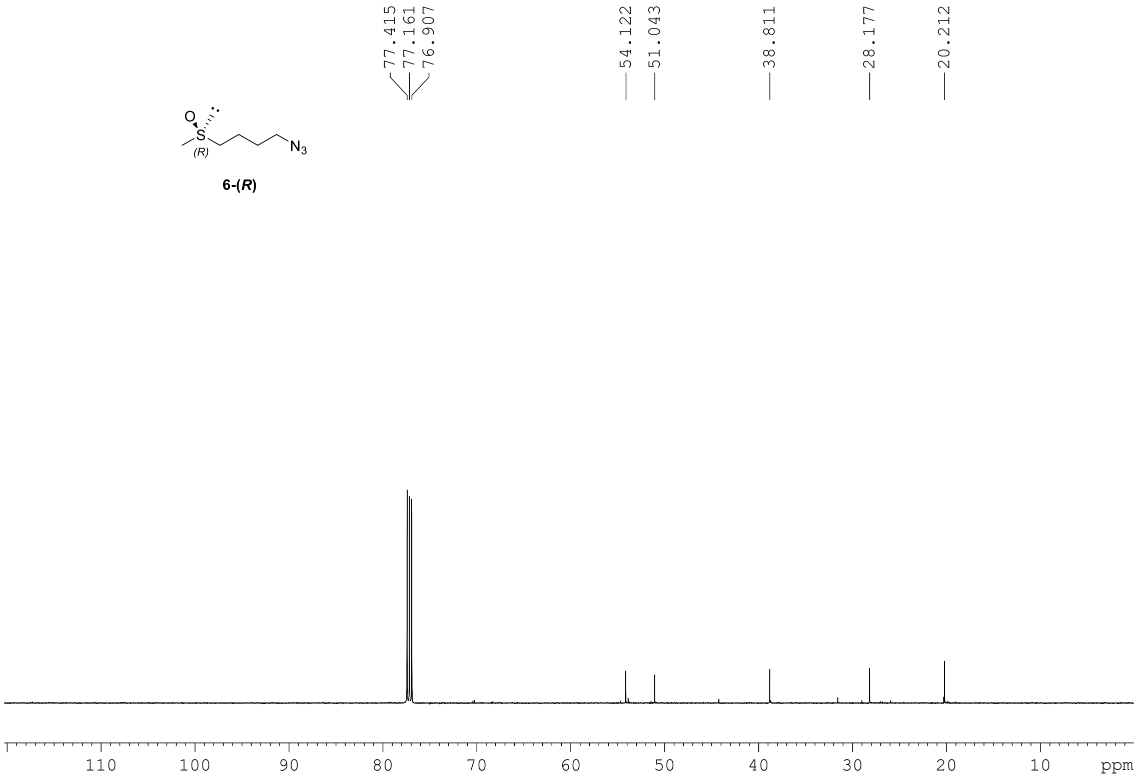

Figure S13. ^{13}C-NMR of (*R*)-(-)-1-Azido-4-(methylsulfinyl)-butane (**6-(*R*)**)

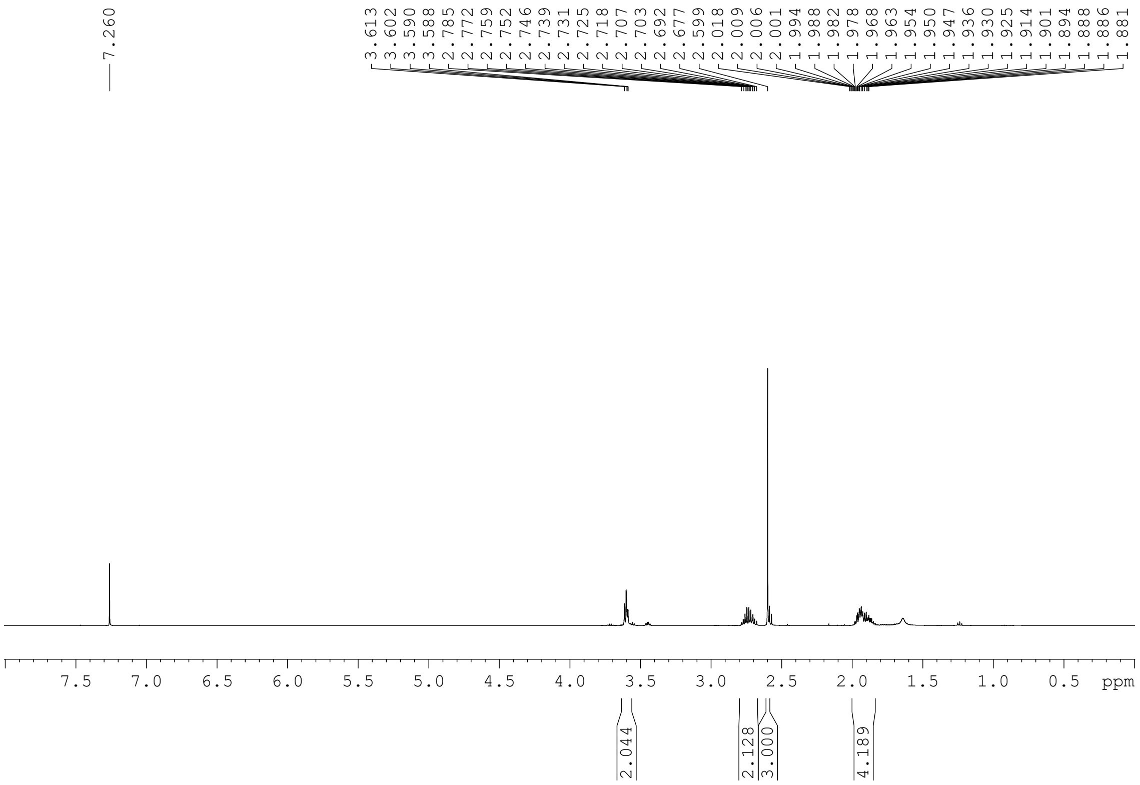

Figure S14. ^{1}H-NMR of (*R*)-(−)-1-Isothiocyanato-4-(butylsulfinyl)-butane (***R*-sulforaphane**)

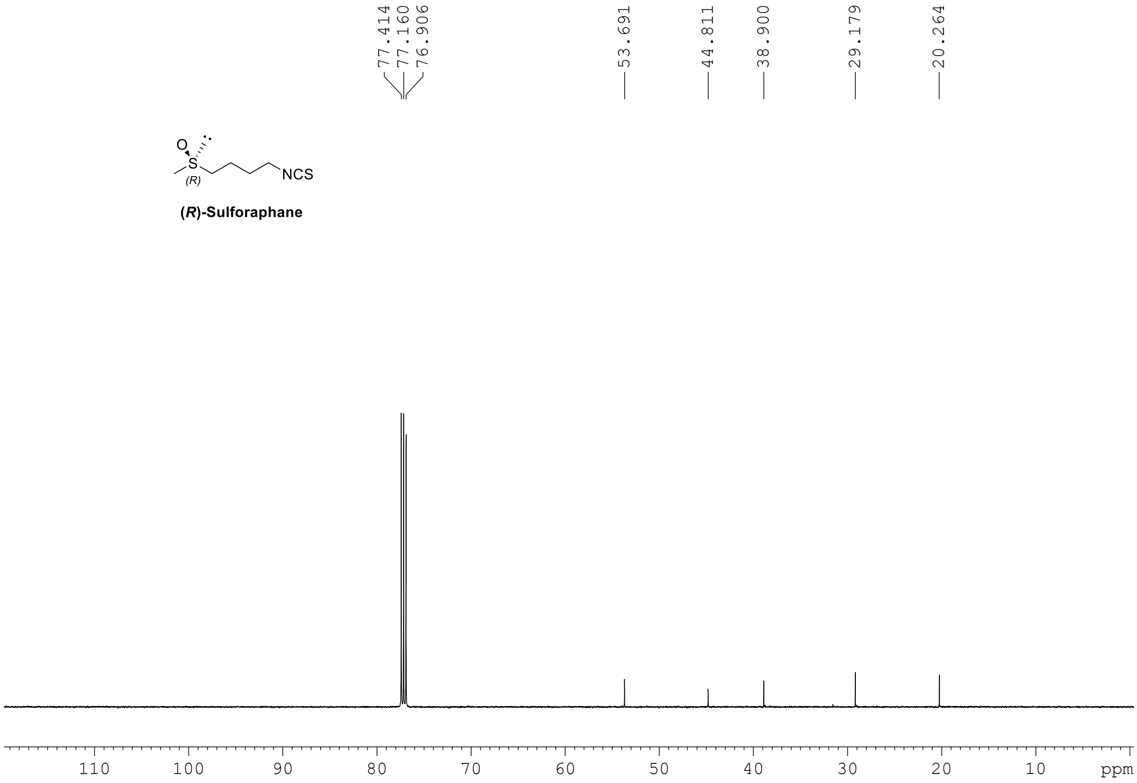

Figure S15. ^{13}C-NMR of (*R*)-(−)-1-Isothiocyanato-4-(butylsulfinyl)-butane (***R*-sulforaphane**)
